# Supplementary material for: Reticulate evolution in eukaryotes: Origin and evolution of the nitrate assimilation pathway
Source: PLoS Genet. 2019 Feb 21;15(2):e1007986. doi: 10.1371/journal.pgen.1007986 (PMC6400420; doi:10.1371/journal.pgen.1007986)
Supplement: S2 Supporting information — (PDF) [file pgen.1007986.s002.pdf]

NRT2

Step 1: We ran *hmmsearch* (HMMER version 3.1b1) (Eddy 1998) using the *MFS_1* HMM profile to query euk_db [only non-default software parameters specified]. *MFS_1* sequences were retrieved and submitted to *cdhit* (version 4.6) (Fu et al. 2012) [-c 0.99] to remove repeated / very recent paralogues (i.e. redundant sequences). NRT2 references were aligned with this set using *BLASTP* (version 2.3.0+) (Altschul et al. 1990) [-evalue 1e-5] and the aligned sequences were used to capture sequences in prok_db also using *BLASTP* [-evalue 1e-5, -max_target_seqs 3]. From the aligned sequences of prok_db, we kept only those with a MFS_1 domain according to *hmmsearch*. Proteins captured from euk_db and prok_db were aligned with *MAFFT* (version v7.123b) (Katoh et al. 2002) [mafft-einsi] and the alignment was trimmed with *trimAl* (version v1.4.rev15) (Capella-Gutierrez et al. 2009) using the -gappyout option. Maximum likelihood phylogenetic inference was done using *RAxML* (version 8.2.4) (Stamatakis 2014) with rapid bootstrap analysis (100 replicates), gamma model of rate heterogeneity and LG amino acid substitution matrix. We used the constructed phylogenetic tree to perform a first round to discard sequences unlikely to correspond to eukaryotic NRT2 (Supplementary file 1-Figure 1). This process was assisted by the information obtained through the SwissProt-based annotation: proteins were locally aligned with *BLASTP* against the SwissProt database (Apweiler et al. 2004) (downloaded on July 2016) and annotated on the basis from the best hits of each query. When a clade was composed mainly by eukaryotic sequences annotated as ‘High-affinity nitrate transporter’, it was considered as a *bona fide* eukaryotic NRT2 clade and sequences were kept for the next analyses. Any particular sequences not annotated as NRT2 but consistently branching within a NRT2 clade were also kept. This occurred in very few cases since annotations were highly consistent within the different clades of the tree. Sequences annotated as NRT2 but branching in a clade mostly including sequences that were not annotated as NRT2 were also subsequently re-

evaluated in Step 2. If an eukaryotic sequence branched within a bacterial NRT2 clade, it was not kept and was assigned to the corresponding species as a potentially contaminant NRT2 in supplementary table S1. This included one and two sequences from *Nematostella vectensis* and *Nutomonas longa*, respectively. The *N. vectensis* sequence was found to be located in a small genomic fragment without any gene of eukaryotic ancestry (data not shown). *N. longa* sequences were obtained from transcriptomics data from which we could expect bacterial contamination (Torruella et al. 2015). Sequences that were not discarded were colored in blue in Supplementary file 1-Figure 1.

Step 2: Sequences from Step 1 were used to capture putative NRT2 sequences in prok_db with *BLASTP* [-evalue 1e-5, -max_target_seqs 3] to be used as outgroup. Sequences were aligned with *MAFFT* [mafft-einsi] and trimmed with *trimAl* [-gappyout] and a phylogenetic tree was constructed using *RAxML* with rapid bootstrap analysis (100 replicates), using a gamma model of rate heterogeneity, LG amino acid substitution matrix and considering invariant sites and estimating the empirical frequencies. The model was selected according to BIC criteria with *ProtTest* (version 3.2) (Darriba et al. 2011). All the sequences in the eukaryotic clade (the clade with colored nodes) were then considered as *bona fide* eukaryotic NRT2 (Supplementary file 1-Figure 2).

<u>EUKNR</u>

Step 1: The canonical eukaryotic nitrate reductases are composed by five Pfam domains (Finn et al. 2014): *Oxidored_molyb*, *Mo-co_dimer*, *Cyt-b5*, *FAD_binding_6* and *NAD_binding_1* [*PfamScan*]. Because of miss-annotations or *de facto* gene fission events, we considered as putative EUKNR any protein aligning with the EUKNR reference sequences [*BLASTP*: -evalue 1e-5] and with any of the first two domains according to *hmmsearch*. The selected and non-redundant sequences [*cdhit*: -c 0.99] were aligned

against prok_db to capture those prokaryotic sequences showing similarity [*BLASTP*: -evalue 1e-5, -max_target_seqs 3] and containing at least one of these two domains [*hmmsearch*]. The two sets of sequences retrieved from euk_db and prok_db were aligned with *MAFFT* [mafft-einsi] and trimmed with *trimAl* [-gappyout]. Maximum likelihood phylogenetic inference was carried out using *RAxML* with rapid bootstrap analysis (100 replicates), gamma model of rate heterogeneity and LG amino acid substitution matrix. The clade with the blue-colored branches (Supplementary file 1-Figure 3) includes almost all putative EUKNR according to SwissProt-based annotation as well as some eukaryotic sulfite oxidases (SOX), and hence was kept for Step 2 analysis. The three eukaryotic sequences annotated also as EUKNR present in the red clade (Mvib_comp6938_c0_seq1_m4803, Acas_g17705 and Acas_g11843) were discarded because they branched with bacterial SOX. Indeed, the eukaryotic EUKNR enzyme has never been found in prokaryotes.

Step 2: In order to increase the phylogenetic signal and be able to discriminate between EUKNR and SOX protein families, a phylogenetic tree was constructed with only the sequences that passed all the filters from the first step (same software and parameters). Since the annotation was consistent with the topology of the tree (Supplementary file 1-Figure 4), we kept only sequences of the blue clade (annotated as EUKNR) with the exception of Xtro_ENSXETP00000063222 and Isca_XP_002416205 because their Pfam domains are unrelated to EUKNR (*Solute_trans_a* and *V-set*, respectively). Aory_CADAORAT00009620, in the red clade, was also conserved because it was annotated as EUKNR.

Step 3: An additional tree was constructed with sequences from the previous step, now adding three SOX sequences (from *Homo sapiens*, *Arabidopsis thaliana* and *Escherichia*

coli; downloaded from NCBI protein) as outgroup. The phylogenetic tree was constructed with the same parameters but also considering invariant sites, in agreement with *ProtTest* results. Aory_CADAORAT00009620 was finally not considered a EUKNR because it branched closer to SOX than to the clade composed by the reference EUKNR sequences (blue clade) (Supplementary file 1-Figure 5).

<u>NAD(P)H-NIR</u>

Step 1: We ran *hmmsearch* [7] over the euk_db using the *NIR_SIR* HMM profile as query, a protein domain characteristic of the nitrite and sulfite reductases. *NIR_SIR* detected sequences were later queried by the NAD(P)H-NIR reference sequences [*BLASTP*: -evalue 1e-5]. Aligned sequences were kept and queried against prok_db [*BLASTP*: -evalue 1e-5, -max_target_seqs 3]. Of the aligned prok_db sequences, we only kept those with *NIR_SIR* signatures [*hmmsearch*]. Redundant sequences were removed [*cdhit*: -c 0.99] before alignment [*MAFFT*: mafft-einsi] and trimming process [*trimAl*: -gappyout]. A phylogenetic tree was constructed using *RAxML* with rapid bootstrap analysis (100 replicates), LG amino acid substitution matrix and a gamma model of rate heterogeneity (Supplementary file 1-Figure 6). As with NRT2 (SwissProt-based annotation, inspection of the tree topology and evaluation of potential contamination) was used to identify the putative NAD(P)H-NIR family members (blue branches, see Supplementary file 1-Figure 6).

Step 2: Sequences selected from the previous step were used to search similar sequences in prok_db [*BLASTP*: -evalue 1e-5 -max_target_seqs 3], which were later on aligned with the *NIR_SIR* HMM [*hmmsearch*]. *NIR_SIR* prok_db sequences were aligned with those from Step 1 using *MAFFT* [mafft-einsi] after removing redundancy [*cdhit*: -c 0.99]. Alignment was automatically trimmed with *trimAl* [-gappyout] and a phylogenetic tree was

constructed using *RAxML* with rapid bootstrap analysis (100 replicates), LG amino acid substitution matrix and a gamma model of rate heterogeneity considering invariant sites. The model was selected in agreement with *ProtTest* results. The tree was visualized and nodes were colored using the same criteria as before (Supplementary file 1-Figure 7). We considered all the eukaryotic sequences as *bona fide* NAD(P)H-NIR because they formed a monophyletic clade.

<u>Fd-NIR</u>

Step 1: The same protocol as with NAD(P)H-NIR was used to capture potential Fd-NIR orthologues and to construct a preliminary tree, using the *NIR_SIR* HMM and the Fd-NIR reference sequences (Supplementary file 1-Figure 8). Proteins from different sulfite and nitrite reductase families were captured in different clades (Supplementary file 1-Figure 8, see figure legend). Sequences from the green clade, which included all the eukaryotic sequences unlikely to correspond to prokaryotic contamination, were kept.

Step 2: Sequences maintained from the preliminary step were used as query to search for similar sequences in prok_db sequences [*BLASTP*: -evalue 1e-5 -max_target_seqs 3] with the NIR_SIR domain [*hmmsearch*]. Sequences were aligned [*MAFFT*: mafft-einsi] and the alignment was trimmed [*trimAl*: -gappyout] after redundancy was removed [*cdhit*: -c 0.99]. A phylogenetic tree was constructed using *RAxML* with rapid bootstrap analysis (100 replicates), LG amino acid substitution matrix and a gamma model of rate heterogeneity with invariant sites. The model was selected according to *ProtTest* results. Again, as with NAD(P)H-NIR, all the eukaryotic sequences were considered as Fd-NIR members because they formed a monophyletic clade (Supplementary file 1-Figure 9).

REFERENCES

Altschul SF, Gish W, Miller W, Myers EW, Lipman DJ. 1990. Basic local alignment search tool. J. Mol. Biol. 215:403–410.

Apweiler R, Bairoch A, Wu CH, Barker WC, Boeckmann B, Ferro S, Gasteiger E, Huang H, Lopez R, Magrane M, et al. 2004. UniProt: the Universal Protein knowledgebase. Nucleic Acids Res. 32:D115–D119.

Capella-Gutierrez S, Silla-Martinez JM, Gabaldon T. 2009. trimAl: a tool for automated alignment trimming in large-scale phylogenetic analyses. Bioinformatics 25:1972–1973.

Darriba D, Taboada GL, Doallo R, Posada D. 2011. ProtTest 3: fast selection of best-fit models of protein evolution. Bioinformatics 27:1164–1165.

Eddy SR. 1998. Profile hidden Markov models. Bioinformatics 14:755–763.

Finn RD, Bateman A, Clements J, Coggill P, Eberhardt RY, Eddy SR, Heger A, Hetherington K, Holm L, Mistry J, et al. 2014. Pfam: The protein families database. Nucleic Acids Res. 42:D222–D230.

Fu L, Niu B, Zhu Z, Wu S, Li W. 2012. CD-HIT: accelerated for clustering the next-generation sequencing data. Bioinformatics 28:3150–3152.

Katoh K, Misawa K, Kuma K, Miyata T. 2002. MAFFT: a novel method for rapid multiple sequence alignment based on fast Fourier transform. Nucleic Acids Res. 30:3059–3066.

Stamatakis A. 2014. RAxML version 8: a tool for phylogenetic analysis and post-analysis of large phylogenies. Bioinformatics 30:1312–1213.

Torruella G, de Mendoza A, Grau-Bové X, Ruiz-Trillo I. 2015. Phylogenomics Reveals Convergent Evolution of Lifestyles in Close Relatives of Animals and Fungi. Curr. Biol. 25:2404–2410.

Supplementary file 1-Figure 1. Maximum likelihood phylogenetic tree inferred during the process of identification of NRT2 family members in eukaryotes. Statistical support values (100-replicates rapid bootstrap) are shown for all nodes. Eukaryotic sequence names are abbreviated with the four-letter code (see supplementary table S1) and colored according to their major taxonomic group (see panel). Sequences names starting with 'UP-' correspond to prokaryotic sequences. Sequences represented in blue branches were kept for the posterior analyses, while those in red were considered unlikely to be bona fide eukaryotic NRT2 sequences and thus were discarded.

Supplementary file 1-Figure 2. Maximum likelihood phylogenetic tree inferred during the process of identification of NRT2 family members in eukaryotes. Statistical support values (100-replicates rapid bootstrap) are shown for all nodes. Eukaryotic sequence names are abbreviated with the four-letter code (see supplementary table S1) and colored according to their major taxonomic group (see panel). All sequences starting with 'UP-' correspond to prokaryotic sequences. All the eukaryotic sequences present in the tree (blue branches) were considered as bona fide eukaryotic NRT2 family members.

Supplementary file 1-Figure 3. Maximum likelihood phylogenetic tree inferred during the process of identification of putative EUKNR family members in eukaryotes Statistical support values (100-replicates rapid bootstrap) are shown for all nodes. Eukaryotic sequence names are abbreviated with the four-letter code (see supplementary table S1) and colored according to their major taxonomic group (see panel). All sequences starting with 'UP-' correspond to prokaryotic sequences. Sequences represented in blue branches were kept for the subsequent analyses, while those in red were considered unlikely to be bona fide eukaryotic EUKNR sequences and thus were discarded (for more details, see Supplementary file 1-Methods).

Supplementary file 1-Figure 4. Maximum likelihood phylogenetic tree inferred during the process of identification of EUKNR family members in eukaryotes. Statistical support values (100-replicates rapid bootstrap) are shown in all nodes. Eukaryotic sequence names are abbreviated with the four-letter code (see supplementary table S1) and colored according to their major taxonomic group (see panel). All sequences starting with 'UP-' correspond to prokaryotic sequences. Sequences represented in blue branches were kept for the posterior analyses, while those in red were considered unlikely to be bona fide eukaryotic EUKNR sequences and thus were discarded.

Supplementary file 1-Figure 5. Maximum likelihood phylogenetic tree inferred during the process of identification of EUKNR family members in eukaryotes. Statistical support values (100-replicates rapid bootstrap) are shown in all nodes. Eukaryotic sequence names are abbreviated with the four-letter code (see supplementary table S1) and colored according to their major taxonomic group (see panel). All sequences starting with 'UP-' correspond to prokaryotic sequences. All the eukaryotic sequences present in the tree (blue branches) were considered as bona fide eukaryotic EUKNR family members (Figure 2) (for more details, see Supplementary file 1-Methods).

Supplementary file 1-Figure 6. Maximum likelihood phylogenetic tree inferred during the process of identification of NAD(P)H-NIR family members in eukaryotes. Statistical support values (100-replicates rapid bootstrap) are shown in all nodes. Eukaryotic sequence names are abbreviated with the four-letter code (see supplementary table S1) and colored according to their major taxonomic group (see panel). All sequences starting with 'UP-' correspond to prokaryotic sequences. Sequences represented in blue branches were kept for the posterior analyses, while those in red were considered unlikely to be bona fide eukaryotic NRT2 sequences and thus were discarded. The four branches colored in yellow correspond to NAD(P)H-NIR sequences identified in eukaryotic proteomes but that are likely bacterial contamination. They were not considered as bona fide eukaryotic NAD(P)H-NIR (Figure 2) and hence were annotated as putative NAD(P)H-NIR in the corresponding species in see supplementary table S1.

Supplementary file 1-Figure 7. Maximum likelihood phylogenetic tree inferred during the process of identification of NAD(P)H-NIR family members in eukaryotes. Statistical support values (100-replicates rapid bootstrap) are shown in all nodes. Eukaryotic sequence names are abbreviated with the four-letter code (see supplementary table S1) and coloured according to their major taxonomic group (see panel). All sequences starting with 'UP-' correspond to prokaryotic sequences. All the eukaryotic sequences present in the tree (blue branches) were considered as bona fide eukaryotic NAD(P)H-NIR family members.

Supplementary file 1-Figure 8. Maximum likelihood phylogenetic tree inferred during the process of identification of Fd-NIR family members in eukaryotes. Statistical support values (100-replicates rapid bootstrap) are shown in all nodes. Eukaryotic sequence names are abbreviated with the four-letter code (see supplementary table S1) and colored according to their major taxonomic group (see panel). All sequences starting with 'UP-' correspond to prokaryotic sequences. Branches colored in green correspond to those

sequences that were kept for the posterior analyses, while those in other colors were considered unlikely to be bona fide eukaryotic Fd-NIR sequences and thus were discarded. According to SwissProt-based annotation (see Supplementary file 1-Methods), pink and orange clades correspond to sulfite reductases, the red clade correspond to NAD(P)H-NIR sequences and the two yellow clades are composed by sulfite/nitrite reductases. The three eukaryotic sequences present in yellow clades were not even annotated as contaminant Fd-NIR because they are not nitrite reductases according to the annotation.

Supplementary file 1-Figure 9. Maximum likelihood phylogenetic tree inferred during the process of identification Fd-NIR family members in eukaryotes. Statistical support values (100-replicates rapid bootstrap) are shown in all nodes. Eukaryotic sequence names are abbreviated with the four-letter code (see supplementary table S1) and colored according to their major taxonomic group (see panel). All sequences starting with 'UP-' correspond to prokaryotic sequences. All the eukaryotic sequences present in the tree (blue branches) were considered as bona fide eukaryotic Fd-NIR family.

Supplementary file 1-figure1

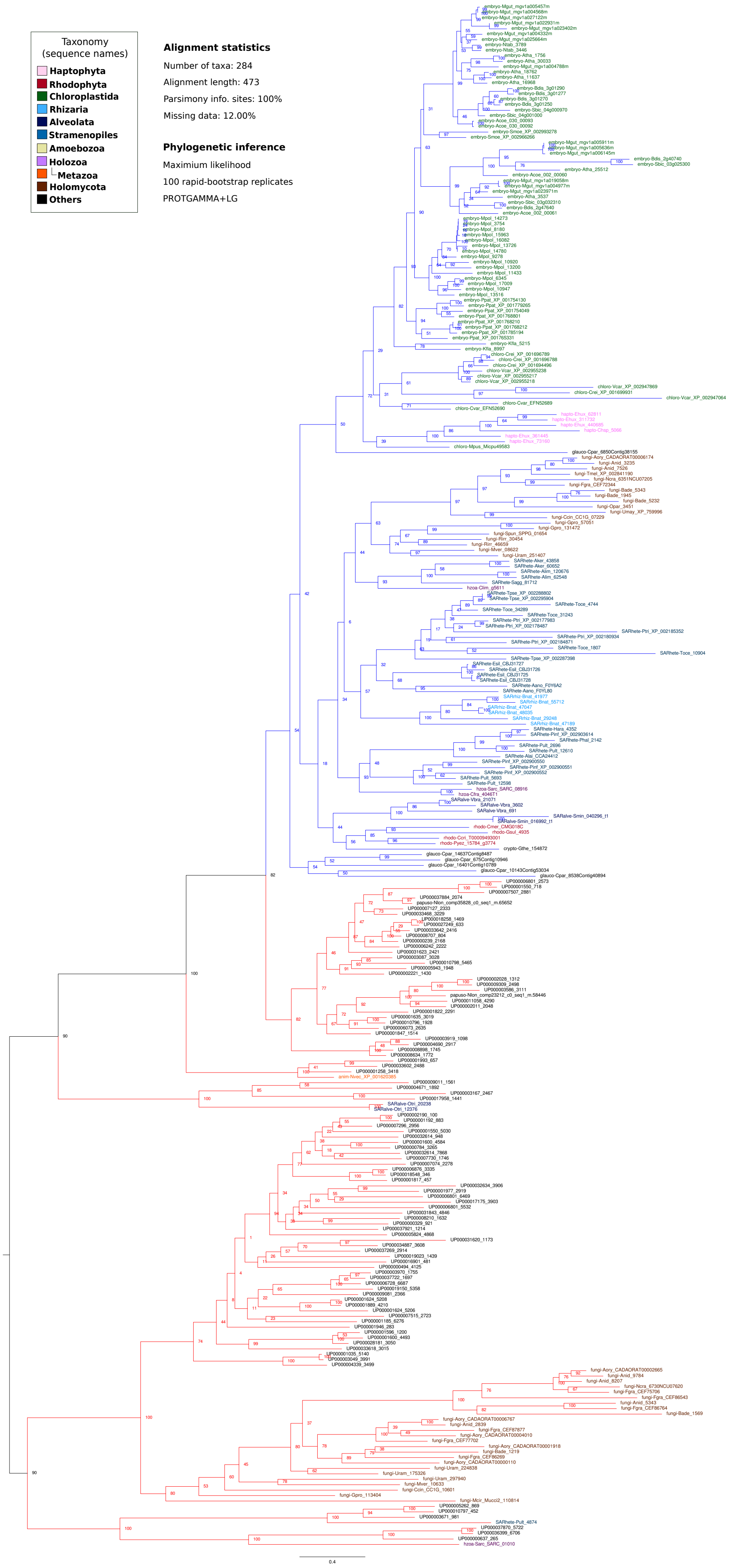

Supplementary file 1-figure2

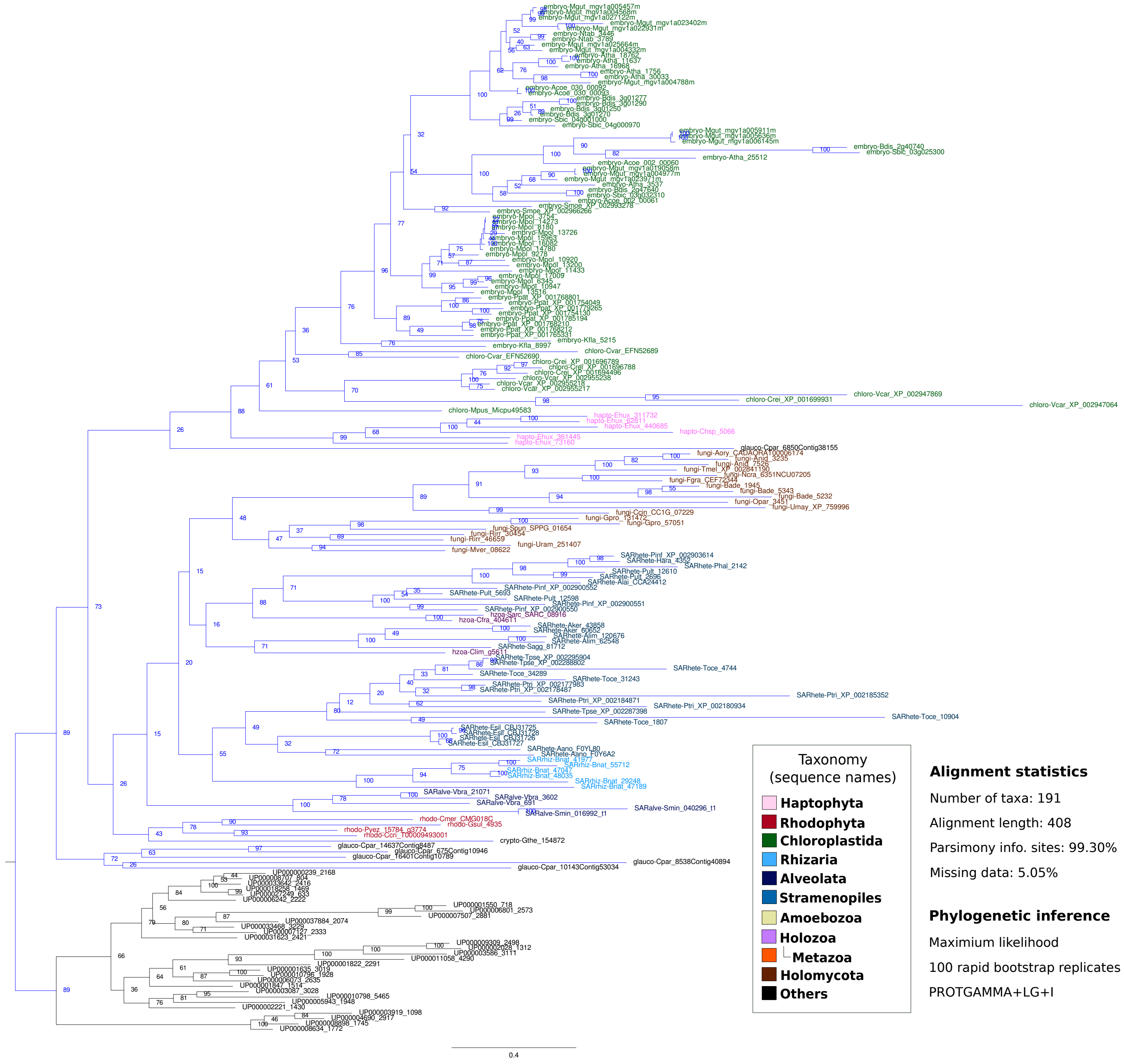

Supplementary file 1-figure3

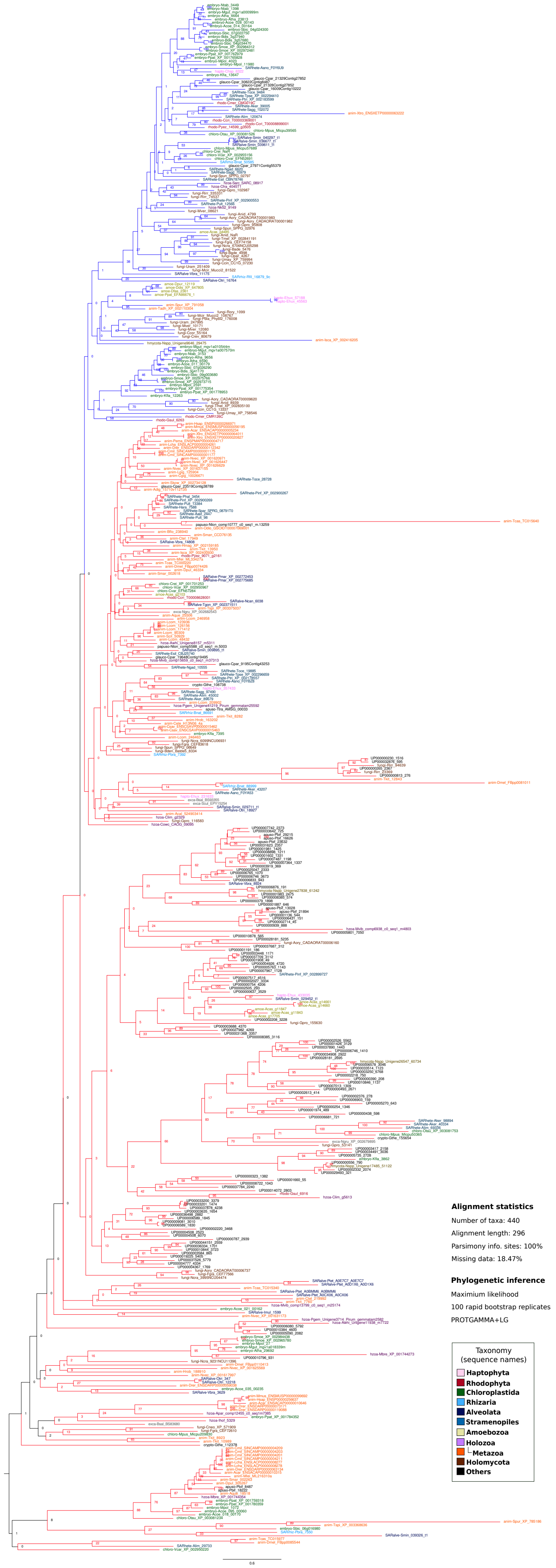

Supplementary file 1-figure4

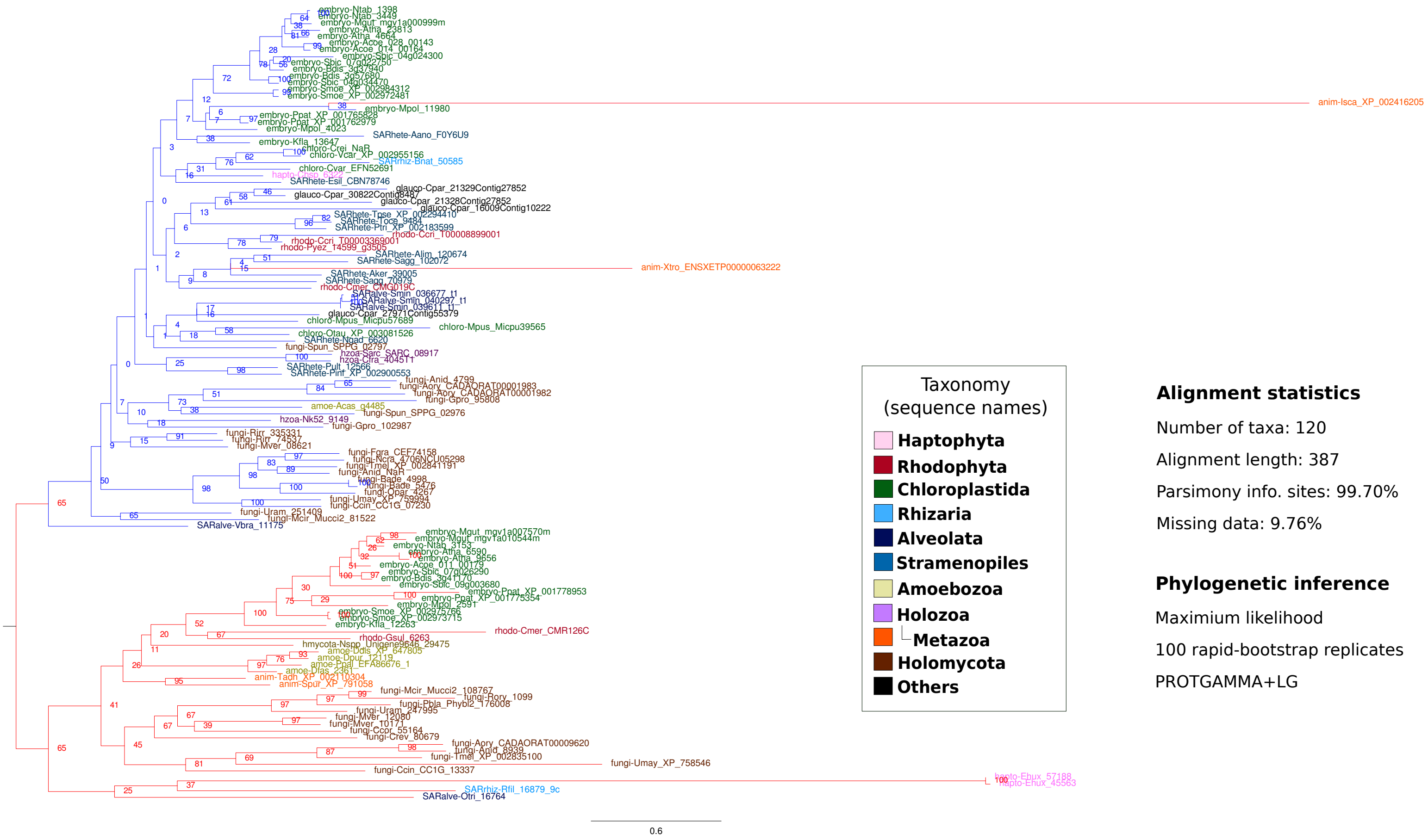

Taxonomy (sequence names)

- Haptophyta
- Rhodophyta
- Chloroplastida
- Rhizaria
- Alveolata
- Stramenopiles
- Amoebozoa
- Holozoa
  - Metazoa
- Holomycota
- Others

Alignment statistics

Number of taxa: 120

Alignment length: 387

Parsimony info. sites: 99.70%

Missing data: 9.76%

Phylogenetic inference

Maximium likelihood

100 rapid-bootstrap replicates

PROTGAMMA+LG

Supplementary file 1-figure5

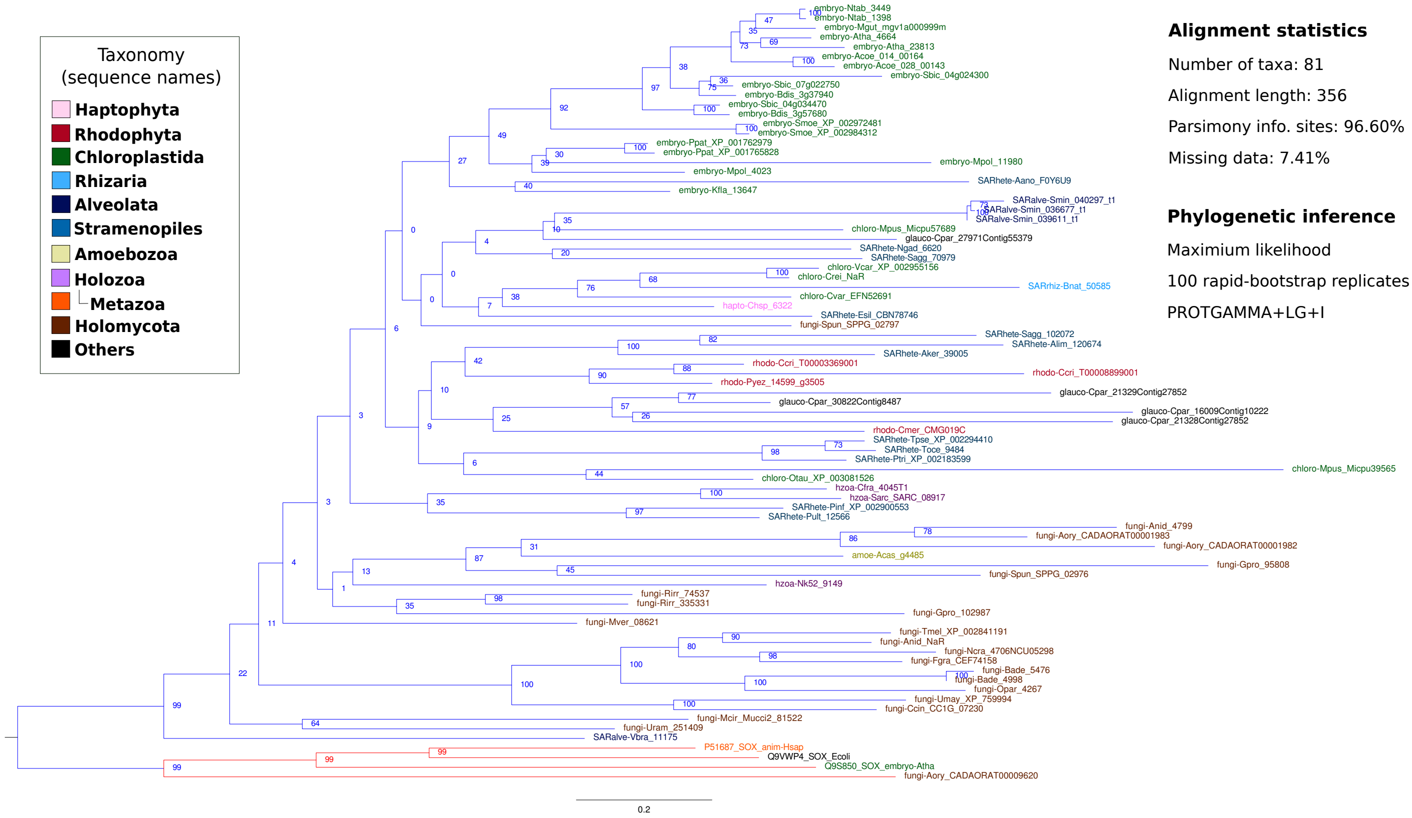

Alignment statistics

Number of taxa: 81

Alignment length: 356

Parsimony info. sites: 96.60%

Missing data: 7.41%

Phylogenetic inference

Maximium likelihood

100 rapid-bootstrap replicates

PROTGAMMA+LG+I

Supplementary file 1-figure6

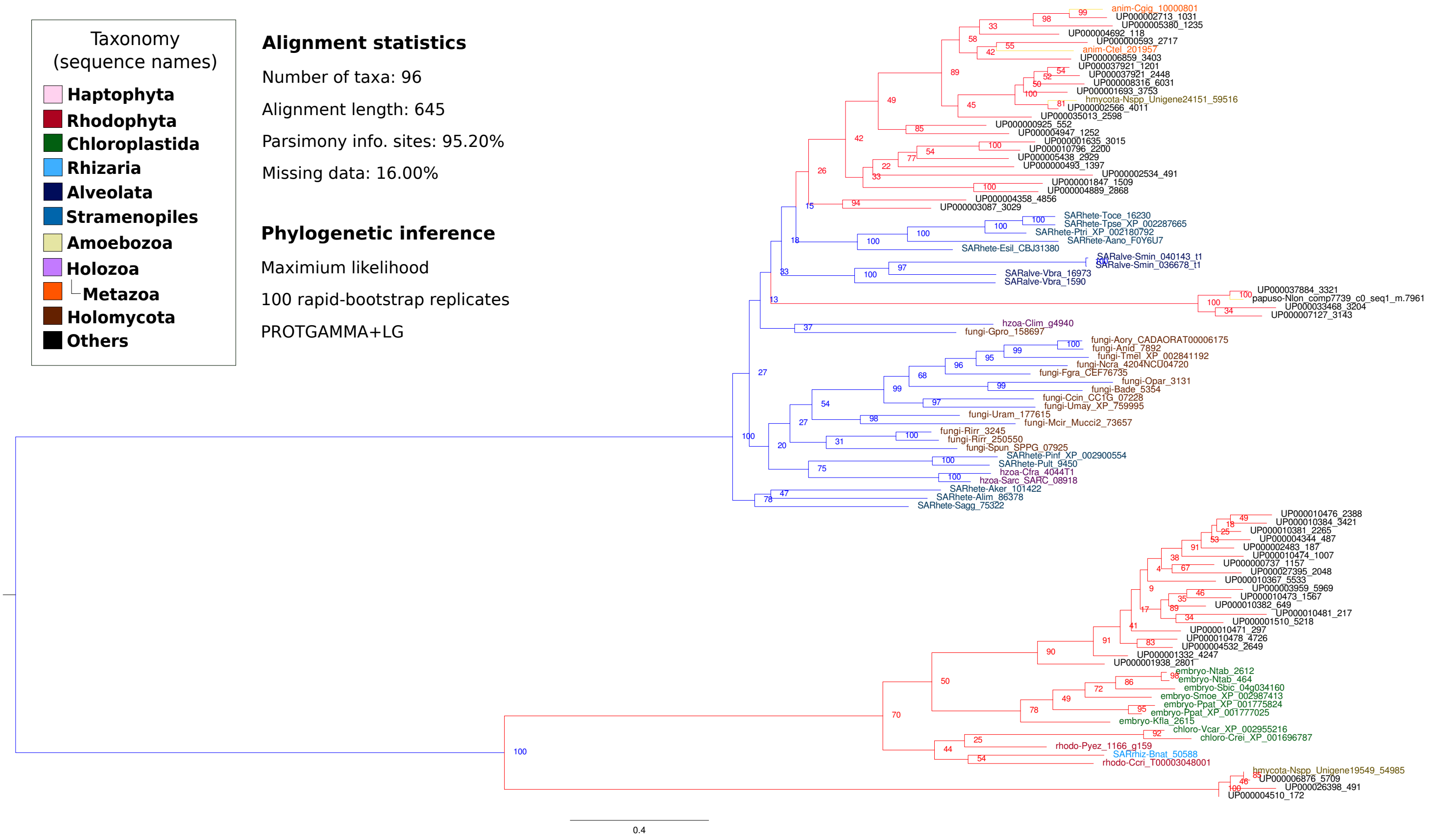

Supplementary file 1-figure7

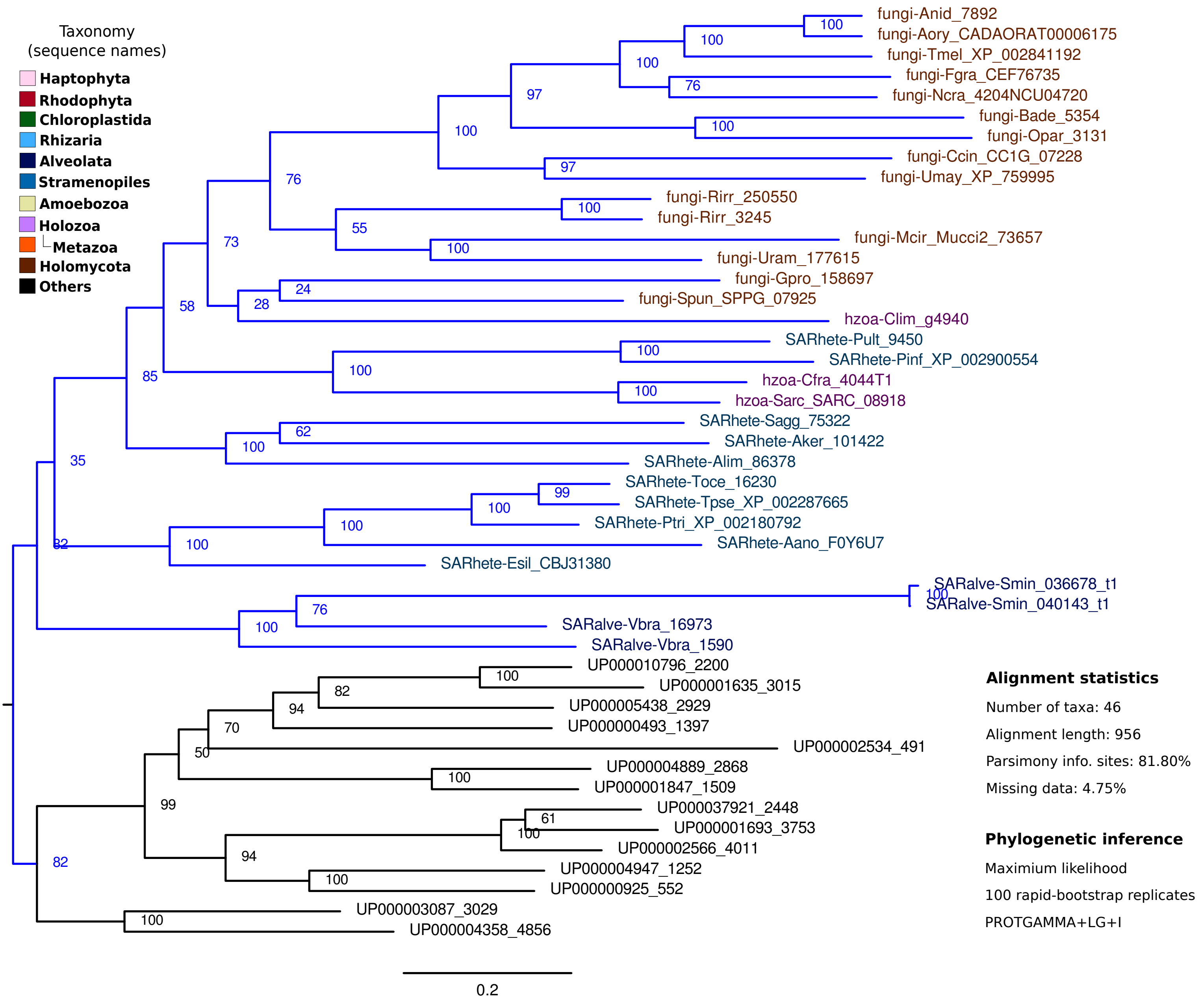

Supplementary file 1-figure8

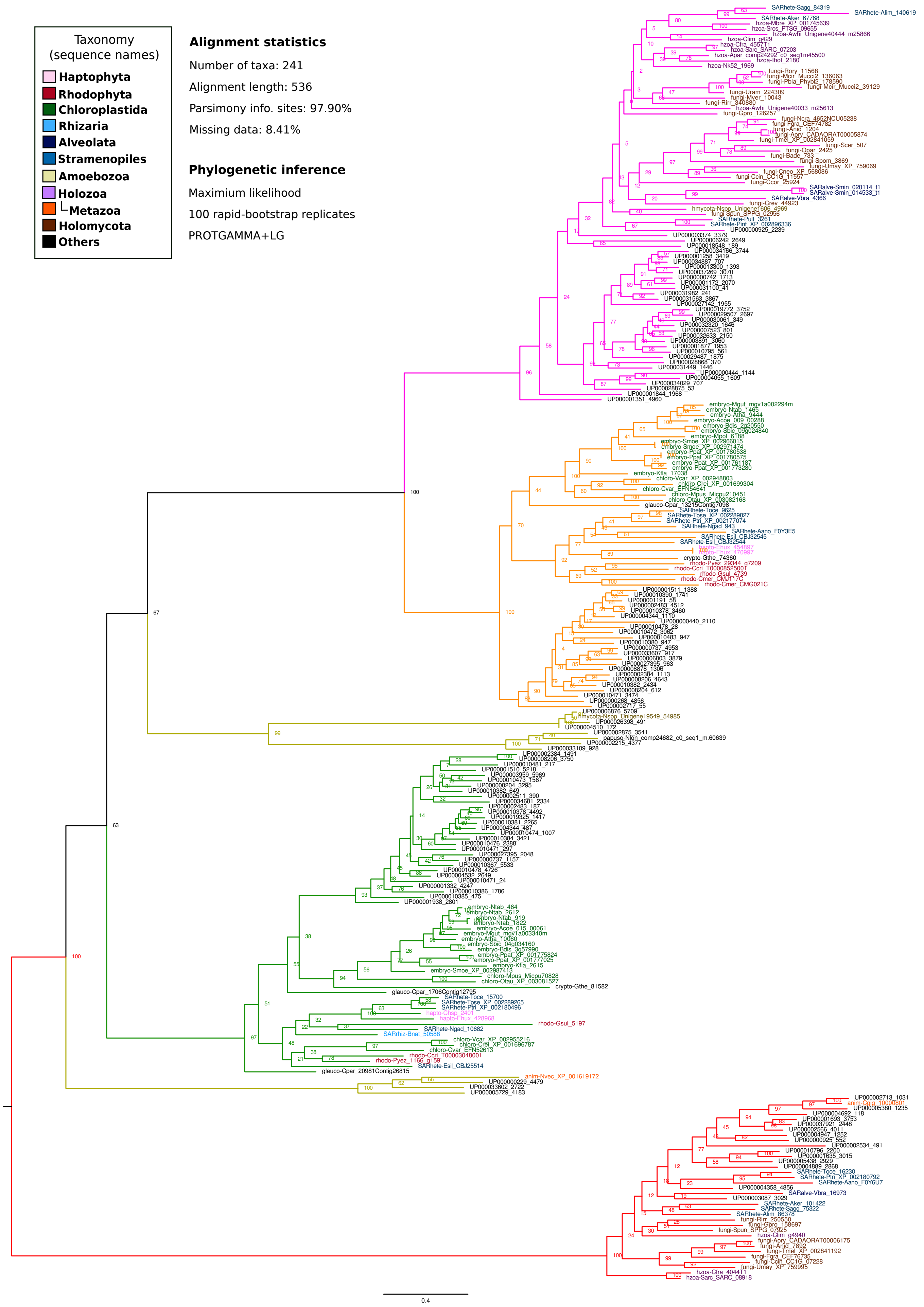

Supplementary file 1-figure9

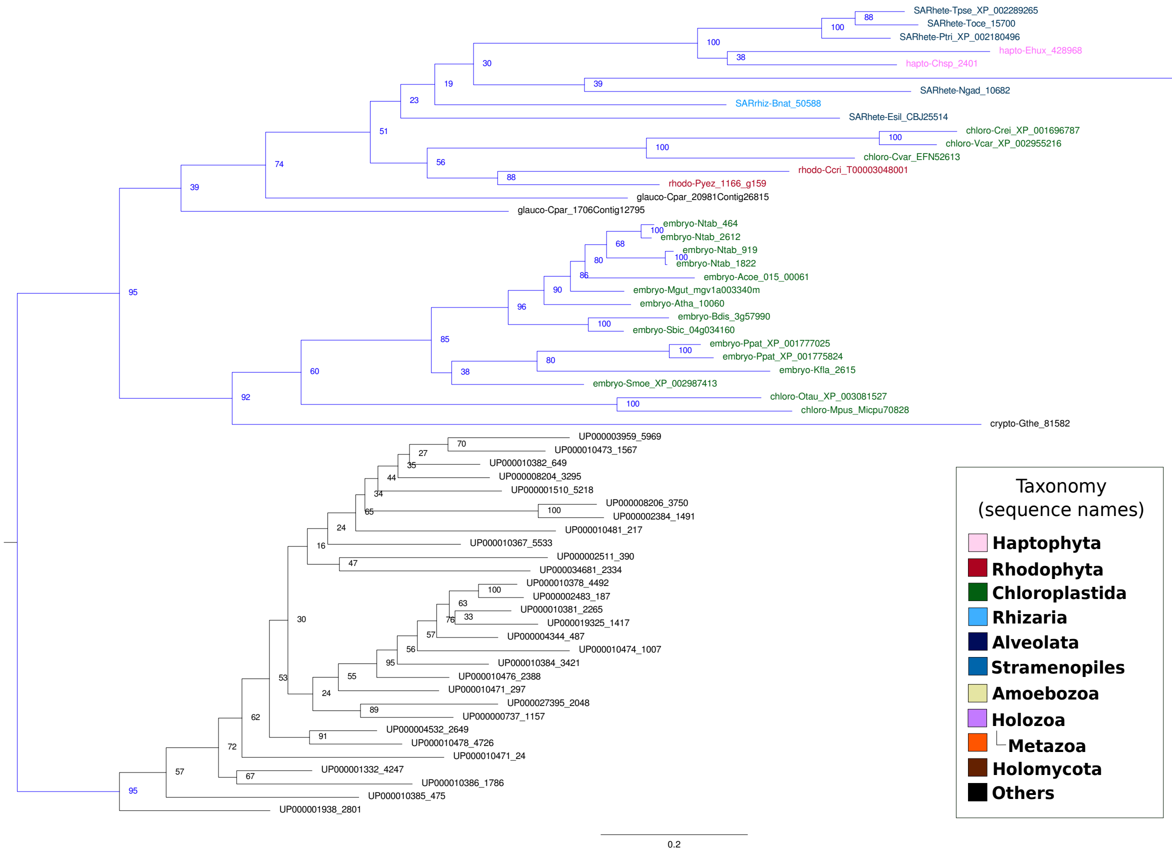

Alignment statistics

Number of taxa: 61

Alignment length: 498

Parsimony info. sites: 81.90%

Missing data: 3.17%

Phylogenetic inference

Maximium likelihood

100 rapid-bootstrap replicates

PROTGAMMA+LG+I
