# Supplementary material for: Reticulate evolution in eukaryotes: Origin and evolution of the nitrate assimilation pathway
Source: PLoS Genet. 2019 Feb 21;15(2):e1007986. doi: 10.1371/journal.pgen.1007986 (PMC6400420; doi:10.1371/journal.pgen.1007986)
Supplement: S4 Supporting information — (PDF) [file pgen.1007986.s004.pdf]

S4 Supporting Information can be downloaded here: doi.org/10.6084/m9.figshare.6462311.v1
