# Supplementary figures and images for: Reticulate evolution in eukaryotes: Origin and evolution of the nitrate assimilation pathway
Source: PLoS Genet. 2019 Feb 21;15(2):e1007986. doi: 10.1371/journal.pgen.1007986 (PMC6400420; doi:10.1371/journal.pgen.1007986)

Supplementary Figure 1

NAPs distribution and pathway completeness

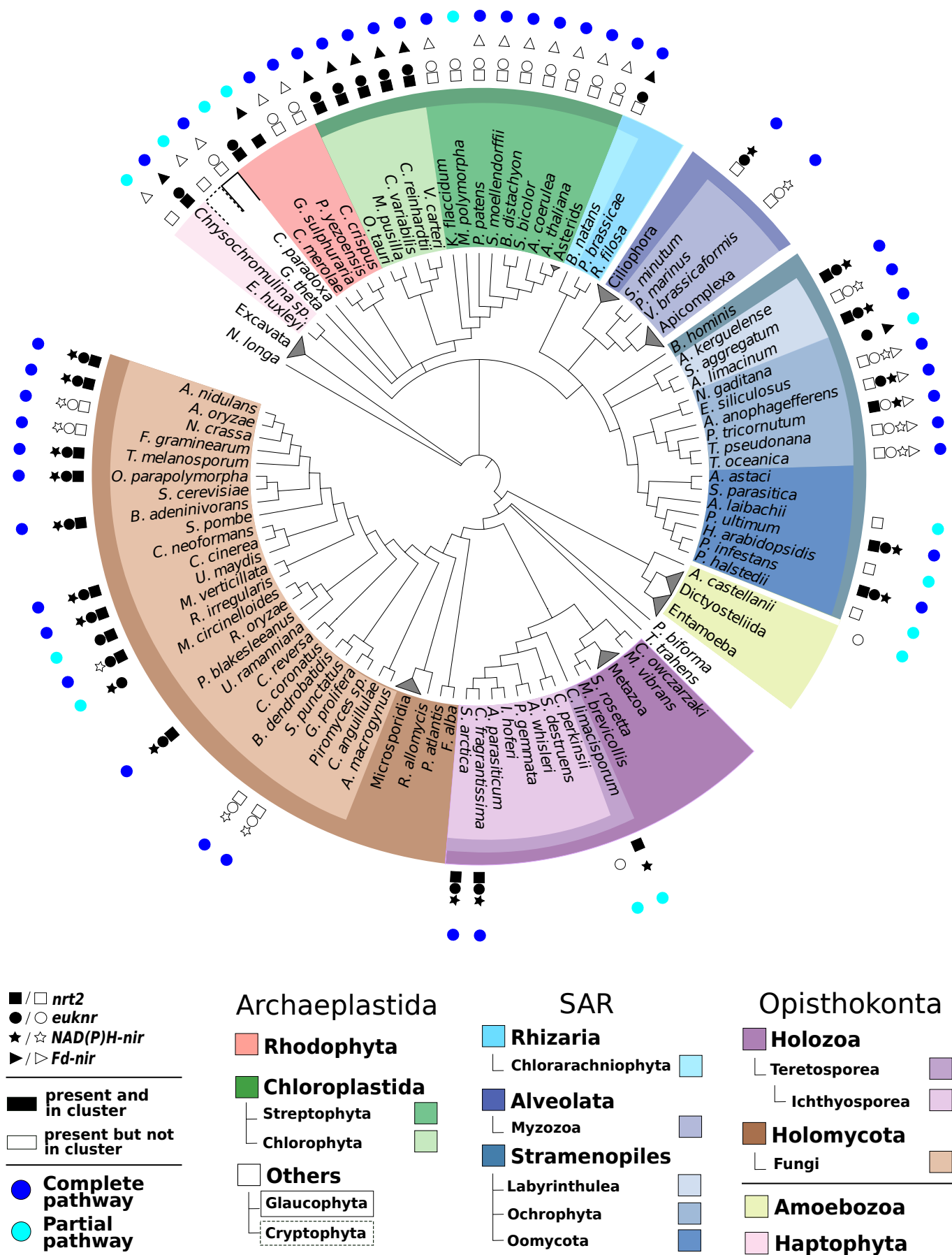

Supplement: S1 Fig — The evolutionary relationships between the sampled species, represented in a cladogram, were constructed from recent bibliographical references (see Materials and methods section). Species names were colored according to the taxonomic groups to which they belong. The presence of each NAP in each taxon is shown with symbols. Black symbols indicate genes that are found within genome clusters of NAP genes. For illustration purposes, some clades of species (e.g. Metazoa) were collapsed into a single terminal leaf. For detailed information about the taxonomic categories and the NAP profiles and NAP cluster status of each species, see Table A in S1 Supporting information. Species are labelled as to whether they include a complete (dark blue circle) or partial pathway (light blue circle). The presence of the pathway was considered complete when the transporter and the two reductase activities (i.e. NRT2, EUKNR and at least 1 of the two NIRs) were detected in the genome. (PDF) [file pgen.1007986.s005.pdf]

Supplementary figure 3

Fd-NIR (euks & proks)

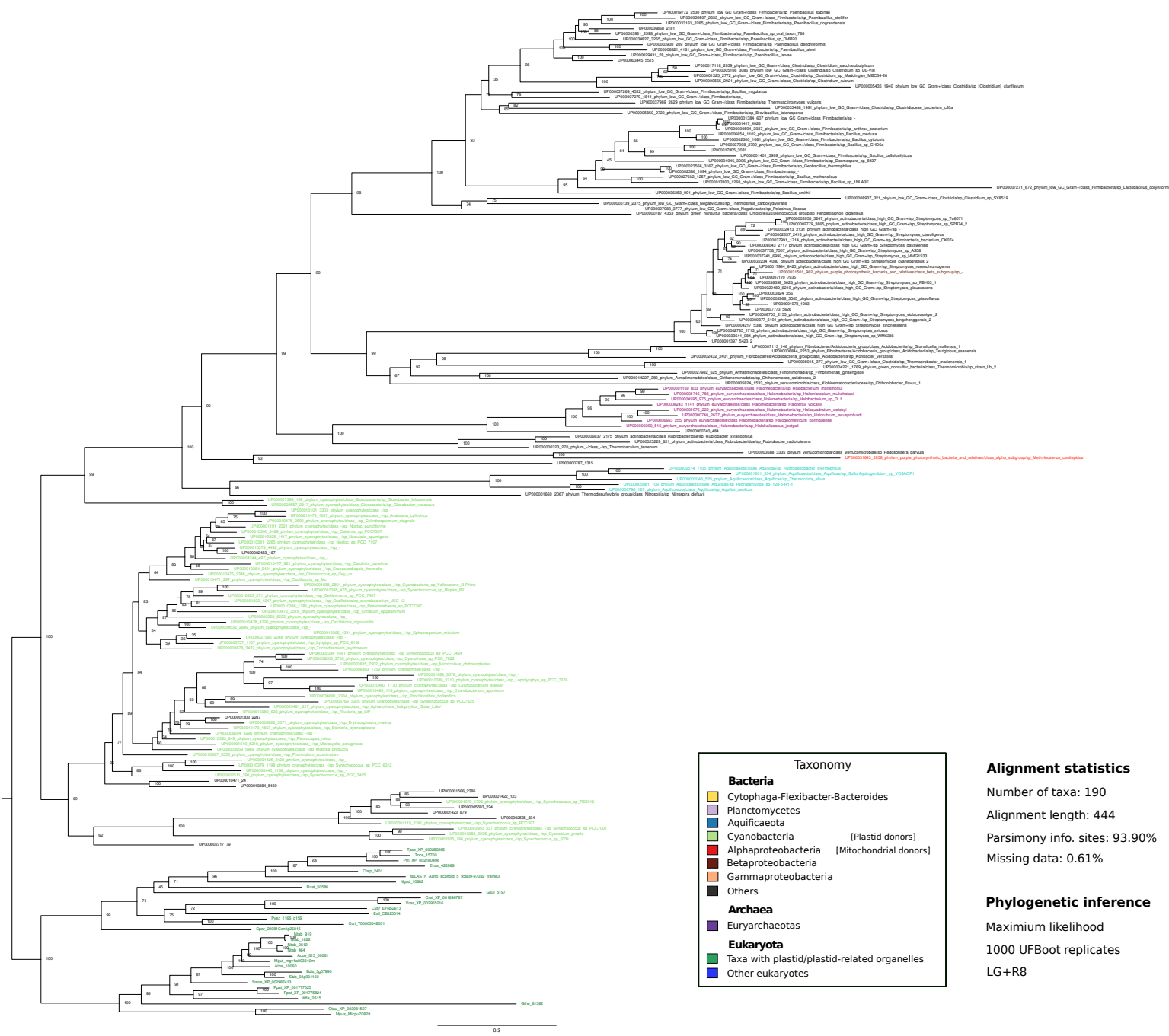

Supplement: S3 Fig — The tree was rooted in the branch that separates the eukaryotic clade from the rest of the tree. Statistical support values (1000-replicates UFBoot) are shown in all nodes. Prokaryotic sequences were colored according to the corresponding phylum or class, while eukaryotes were colored according to whether they contain or not a plastid/plastid-related organelle (see panel). (PDF) [file pgen.1007986.s007.pdf]

Supplementary figure 4

Photosystem II subunit III (euks + proks)

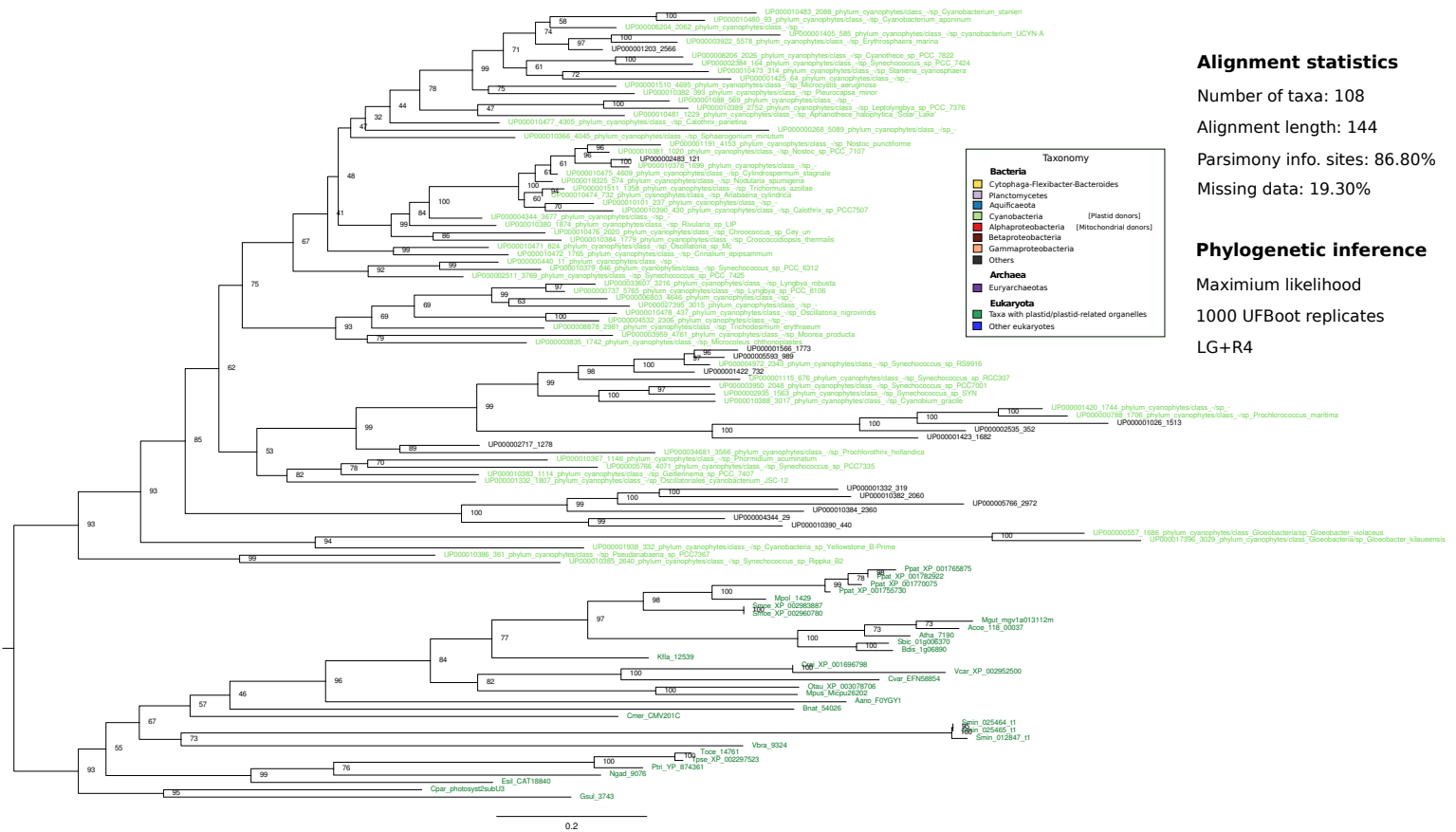

Supplement: S4 Fig — Statistical support values (1000-replicates UFBoot) are shown for all nodes. Prokaryotic sequences were colored according to the corresponding phylum or class, while eukaryotes were colored according to whether they contain or not a plastid/plastid-related organelle (see panel). (PDF) [file pgen.1007986.s008.pdf]

# Supplementary figure 6

## NAD(P)H-NIR (euks + proks)

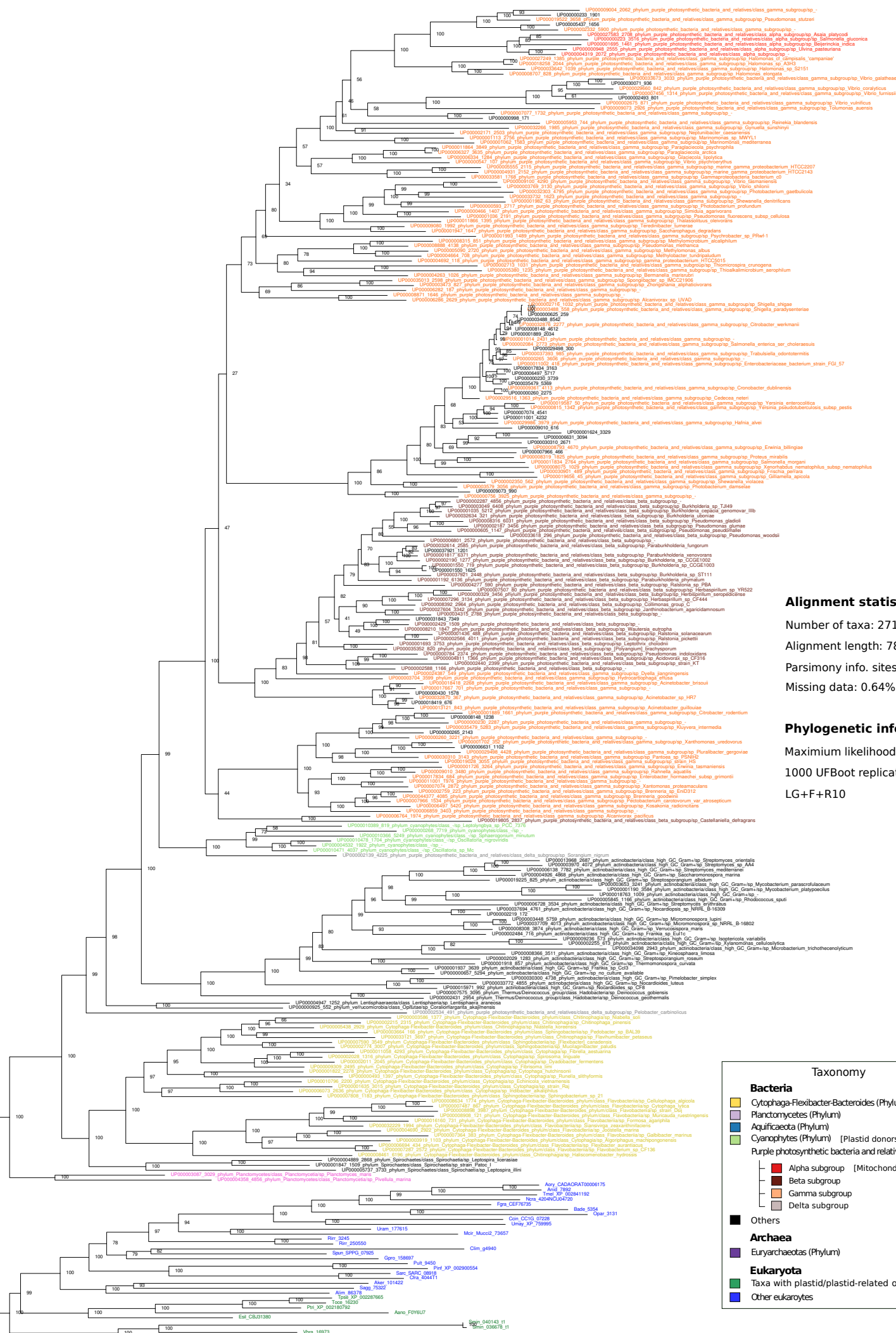

Supplement: S6 Fig — The tree was rooted in the branch that separates the eukaryotic clade from the rest of the tree. Statistical support values (1000-replicates UFBoot) are shown in all nodes. Prokaryotic sequences were colored according to the corresponding phylum or class, while eukaryotes were colored according to whether they contain or not a plastid/plastid-related organelle (see panel). (PDF) [file pgen.1007986.s010.pdf]

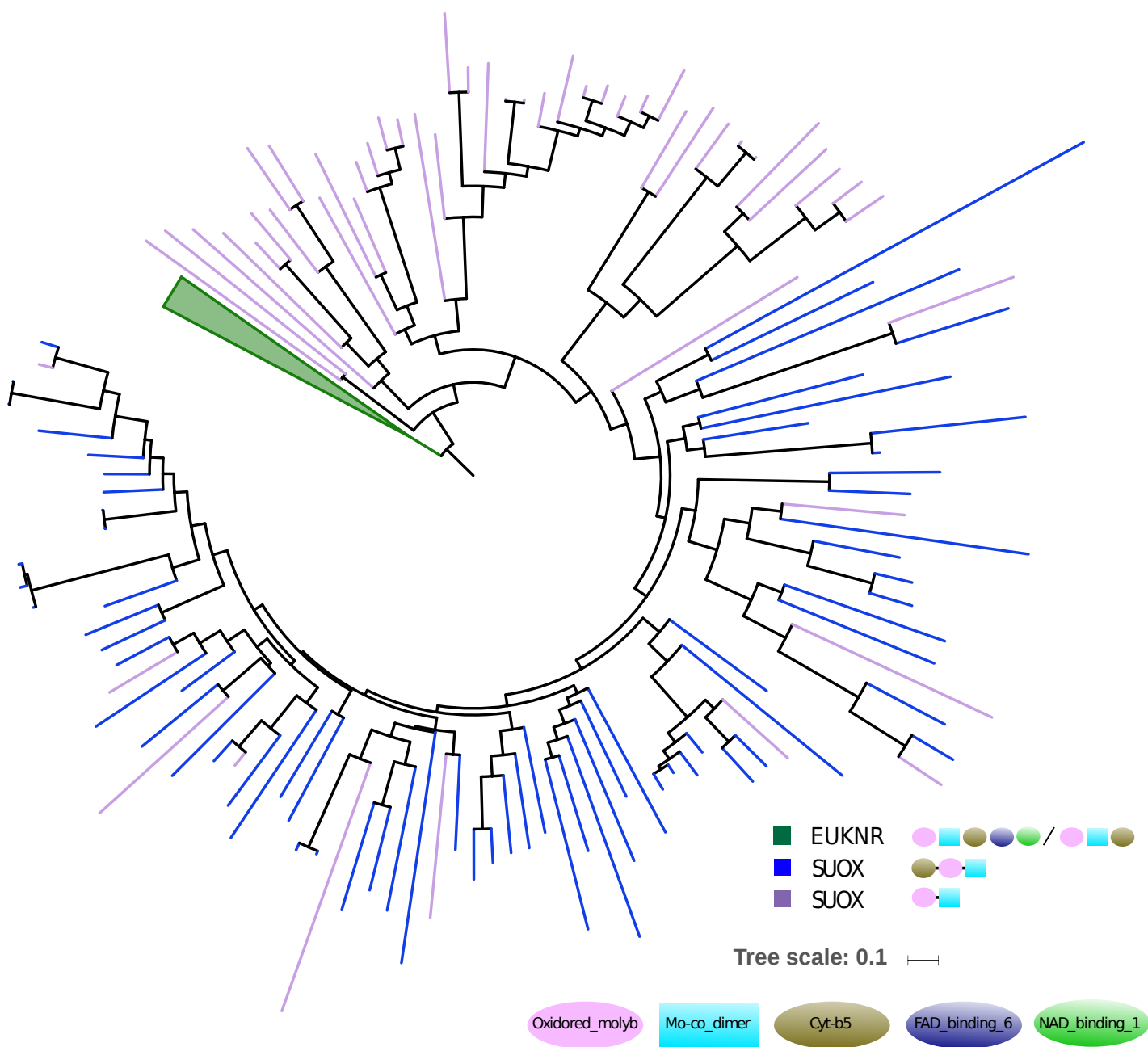

Supplement: S8 Fig — The sulfite oxidases (SUOX) sequences were detected during the EUKNR sequence-similarity network reconstruction process. The topology suggests that EUKNR sequences are more related to SUOX without a Cyt-b5 domain, in agreement with the network results. (PDF) [file pgen.1007986.s012.pdf]

Supplementary figure 9

Fd-NIR (euks)

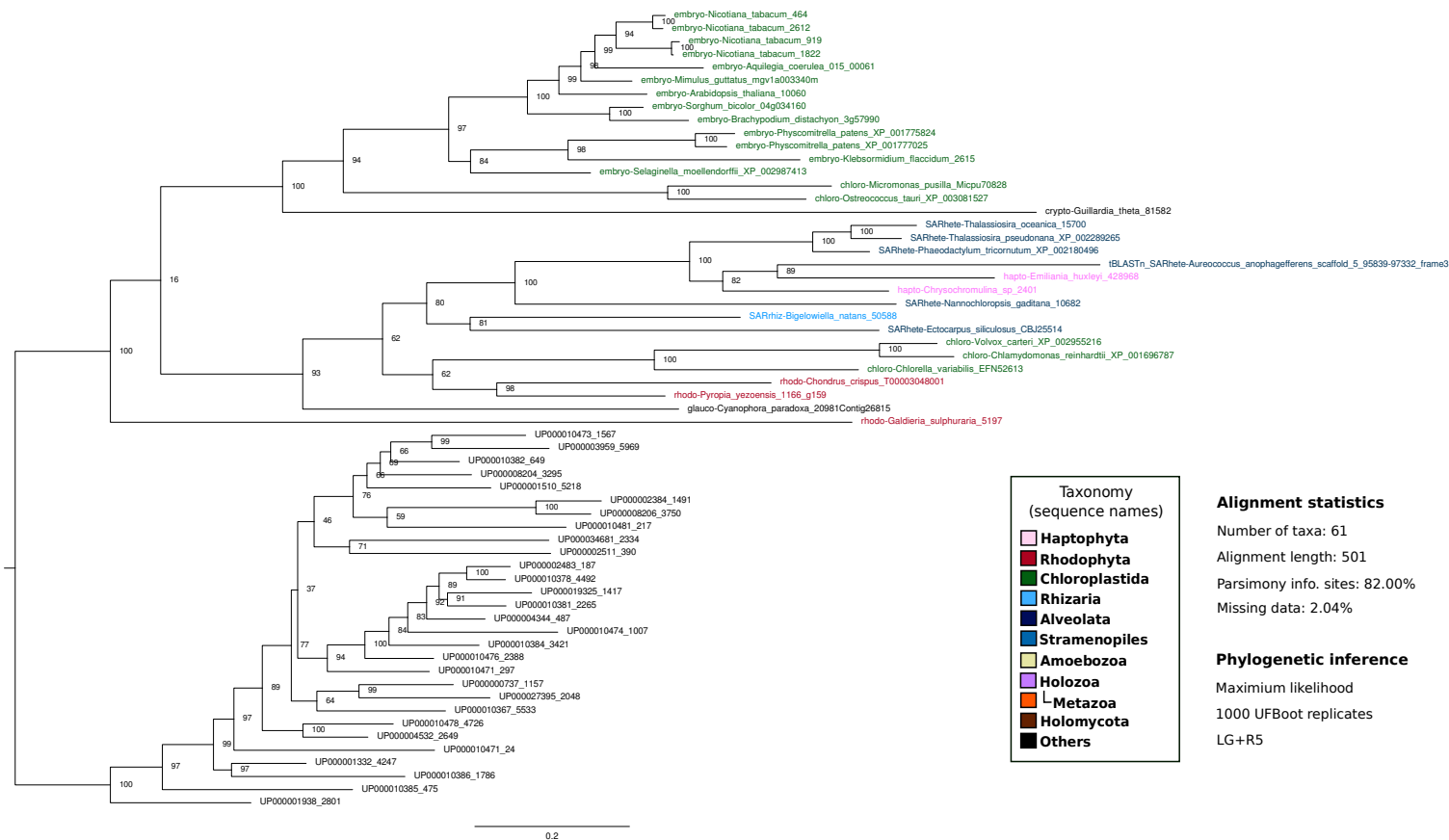

Supplement: S9 Fig — The tree was rooted in the branch that separates the eukaryotic clade from the bacterial. Statistical support values (1000-replicates UFBoot) are shown in all nodes. Eukaryotic sequence names are abbreviated with the four-letter code (see Table A in S1 Supporting information) and colored according to their major taxonomic group (see panel). All sequences starting with 'UP-' correspond to prokaryotic sequences. (PDF) [file pgen.1007986.s013.pdf]

# Supplementary figure 10

## Fd-NIR (euks + MMETSP)

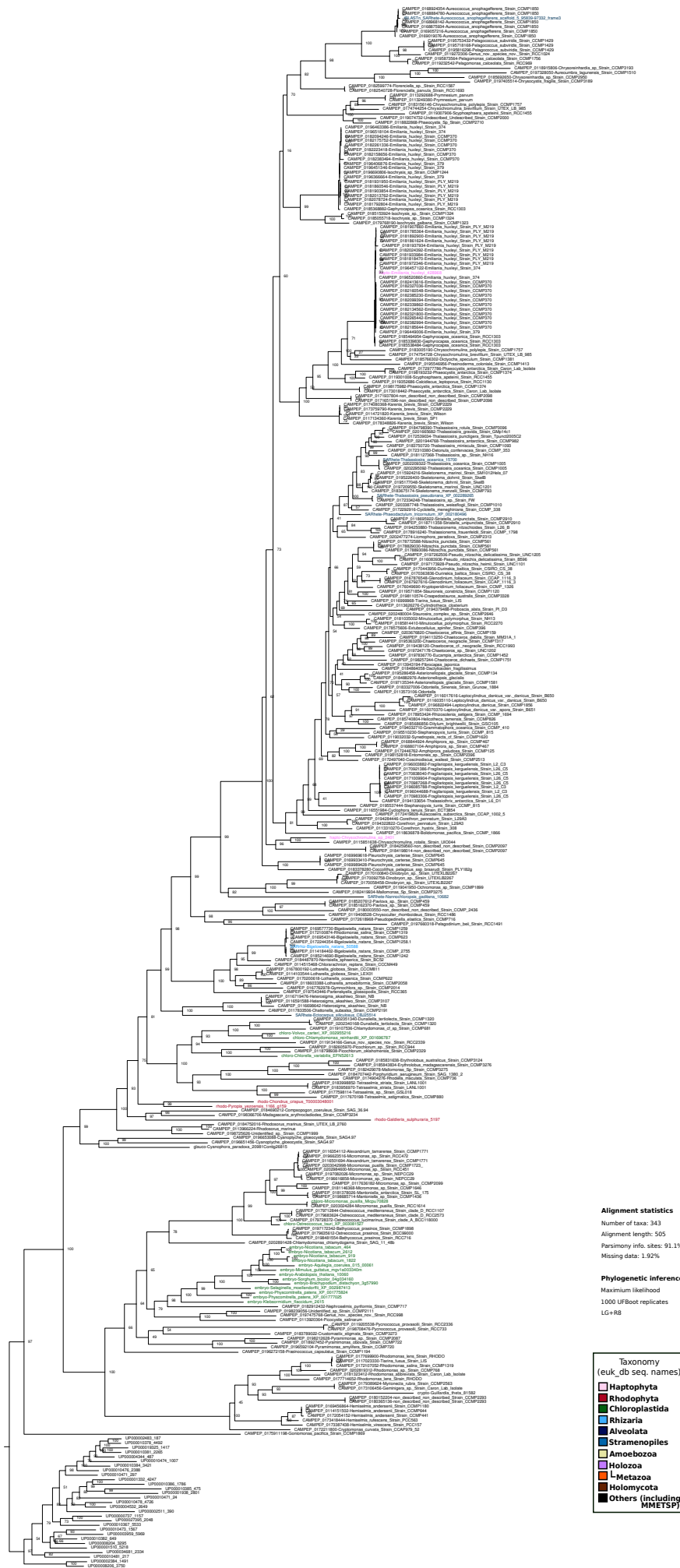

Supplement: S10 Fig — The tree was rooted in the branch that separates the eukaryotic clade from the prokaryotic sequences. Statistical support values (1000-replicates UFBoot) are shown for all nodes. Eukaryotic sequence names from euk_db are abbreviated with the four-letter code (see Table A in S1 Supporting information) and colored according to their major taxonomic group (see panel). Sequences from MMETSP are colored in black. All sequences starting with 'UP-' correspond to prokaryotic sequences. (PDF) [file pgen.1007986.s014.pdf]

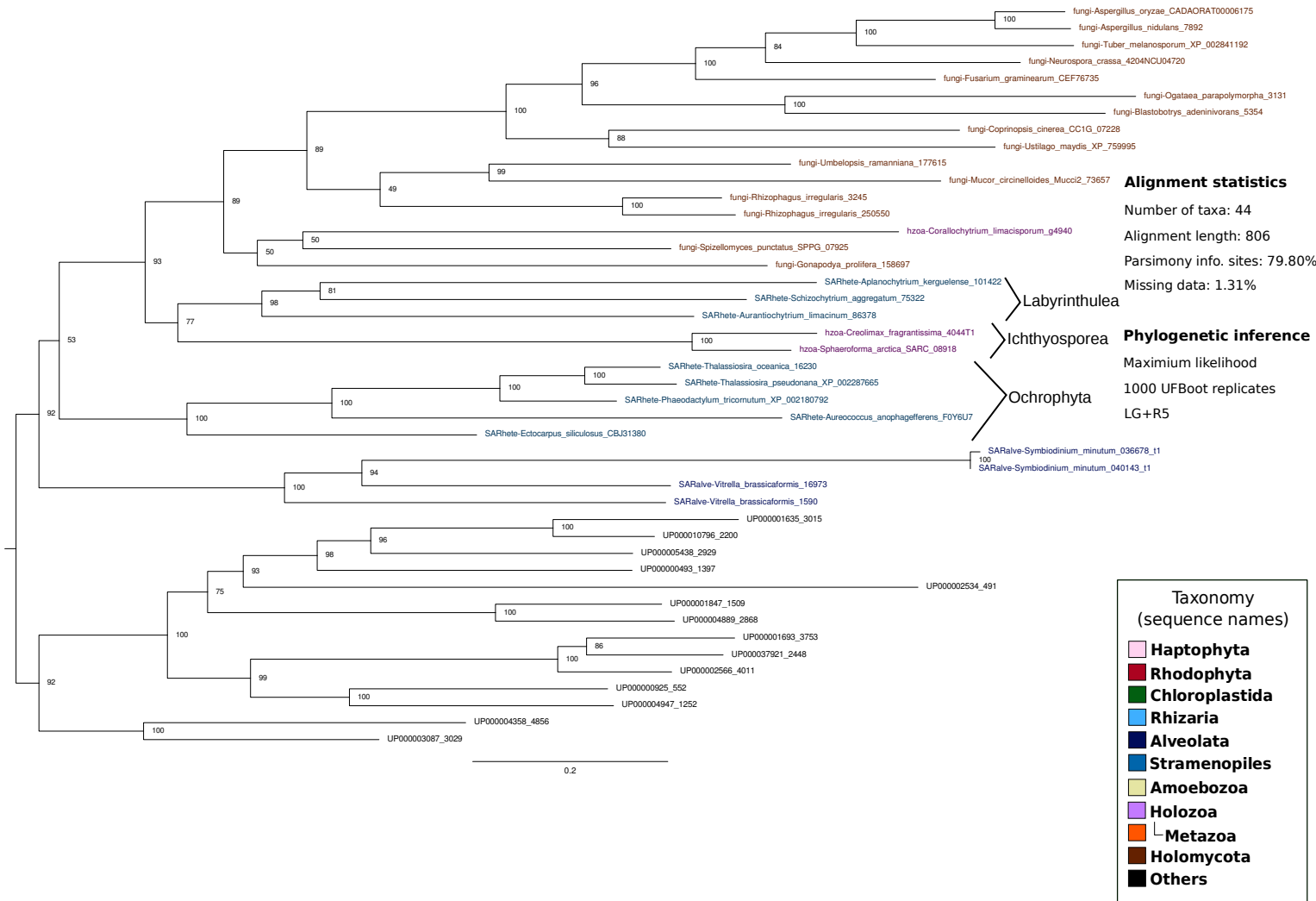

Supplement: S12 Fig — The tree was rooted in the branch that separates the eukaryotic clade from the bacterial sequences, with nodes. Statistical support values (1000-replicates UFBoot) are shown for all nodes. Eukaryotic sequence names are abbreviated with the four-letter code (see Table A in S1 Supporting information) and colored according to their major taxonomic group (see panel). All sequences starting with 'UP-' correspond to prokaryotic sequences. (PDF) [file pgen.1007986.s016.pdf]

Supplementary figure 13

NRT2 (euks)

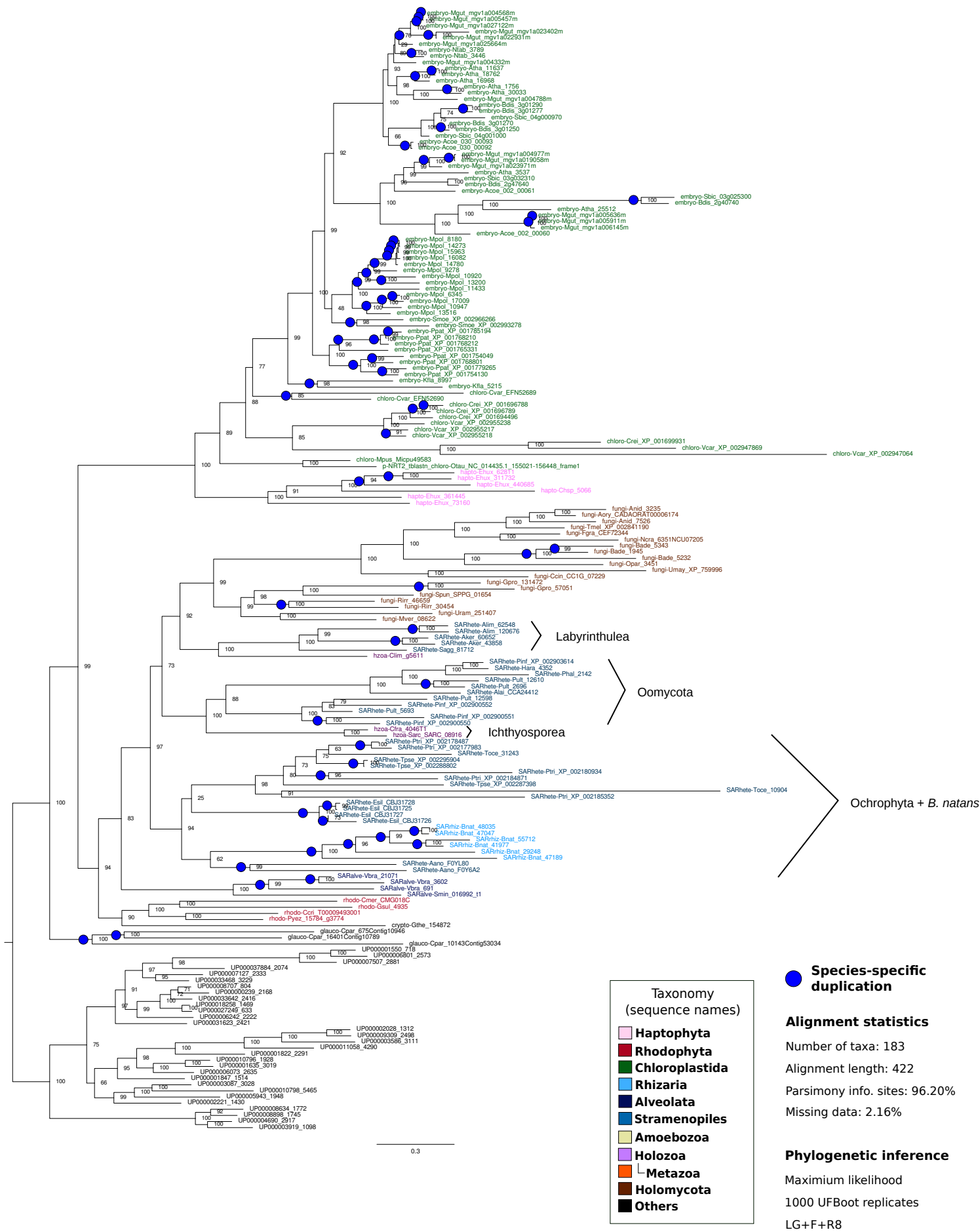

Supplement: S13 Fig — The tree was rooted in the branch that separates the eukaryotic clade from the bacterial sequences. Statistical support values (1000-replicates UFBoot) are shown in all nodes. Eukaryotic sequence names are abbreviated with the four-letter code (see Table A in S1 Supporting information) and colored according to their major taxonomic group (see panel). All sequences starting with 'UP-' correspond to prokaryotic sequences. Nodes with blue circles correspond to species-specific duplication events. (PDF) [file pgen.1007986.s017.pdf]

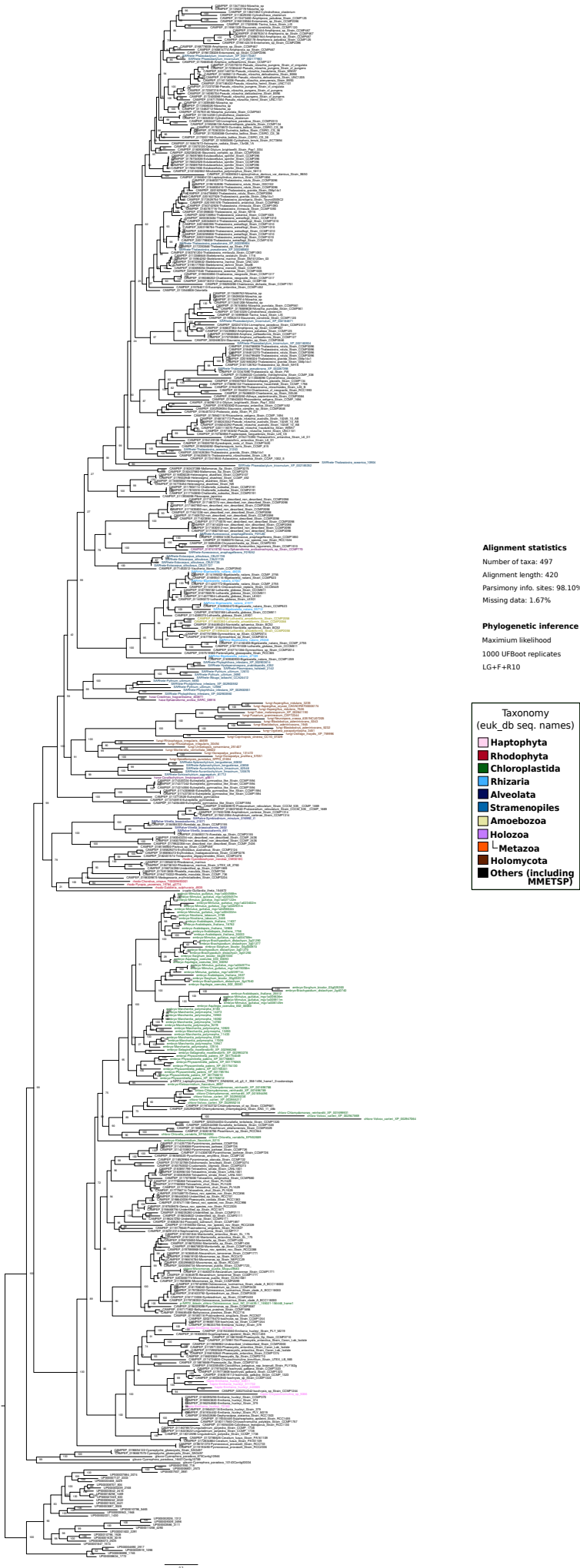

Supplement: S14 Fig — The tree was rooted at the branch that separates the eukaryotic clade from the bacterial sequences. Statistical support values (1000-replicates UFBoot) are shown for all nodes. Eukaryotic sequence names from euk_db are abbreviated with the four-letter code (see Table A in S1 Supporting information) and colored according to their major taxonomic group (see panel). Sequences from MMETSP are colored in black. All sequences starting with 'UP-' correspond to prokaryotic sequences. (PDF) [file pgen.1007986.s018.pdf]

# Hypothetical evolutionary scenarios for NAPs evolution in Opisthokonta and Stramenopiles

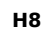

Supplement: S15 Fig — Apart of NAPs, tpmet is also considered, as we found this gene in three NAP clusters (see ‘A tetrapyrrole methylase and the origin of NAPs in Opisthokonta’ Results section). For each scenario, we indicate the branches in which gene transfer, clustering, de-clustering and gene loss events are proposed to have occurred in the evolution of Stramenopiles and Opisthokonta. The proposed donors of the transfers are also indicated. With the exception of Sphaeroforma arctica, Creolimax fragrantissima and Corallochytrium limacisporum, the other species were grouped and the clades were named according to (i) the more inclusive taxonomical category of the taxa represented or (ii) with the four-letter code of the taxa represented (see Table A in S1 Supporting information). For each clade, a symbol of any of the four inspected genes is represented if we detected them in at least one taxa of that clade. Similarly, the largest cluster of TPmet + NAP genes found in each clade is indicated. (PDF) [file pgen.1007986.s019.pdf]

NRT2 (euks, excluding Ichthyosporea)

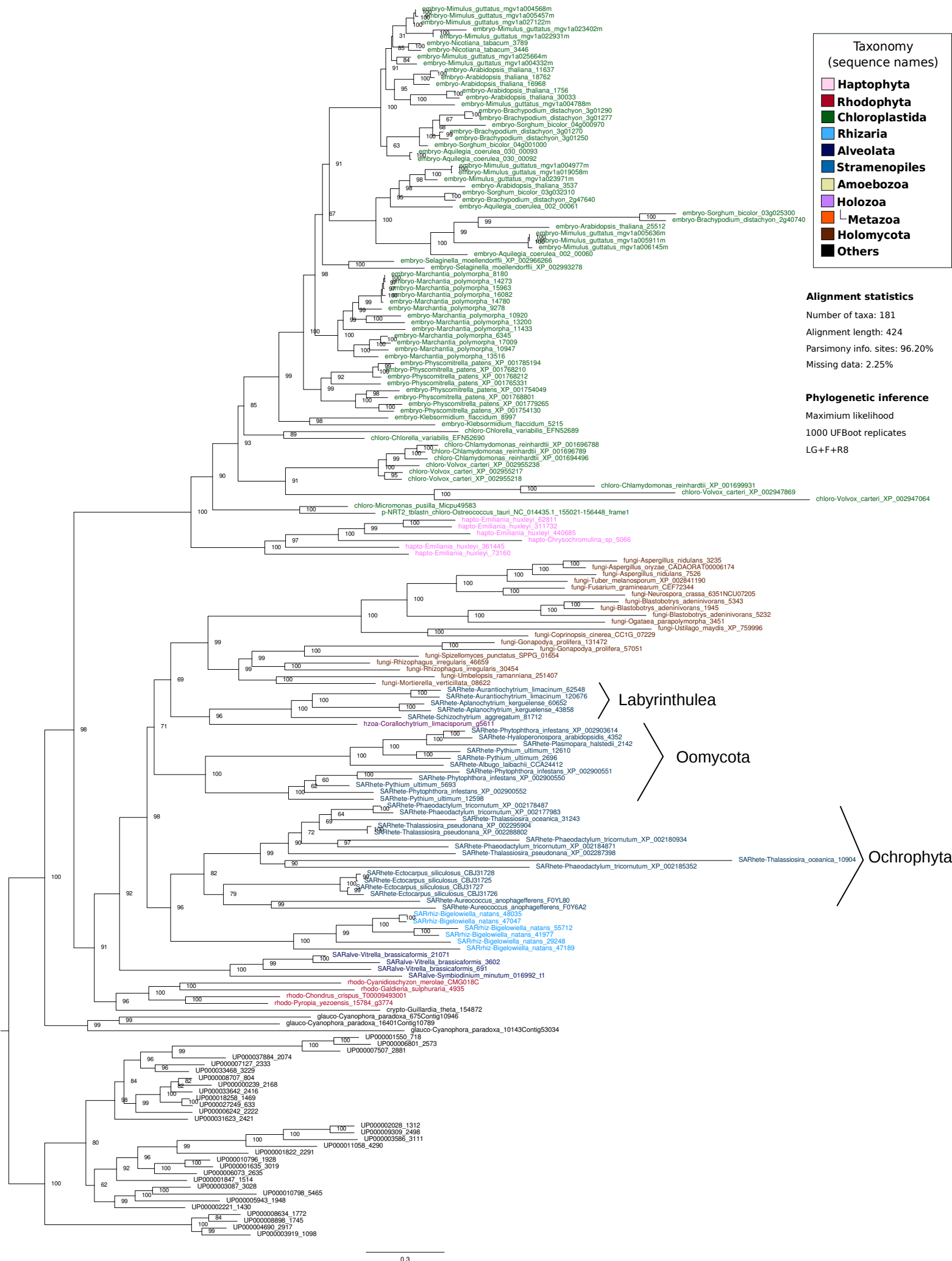

Supplement: S16 Fig — The tree was rooted at the branch that separates the eukaryotic clade from the bacterial sequences. Statistical support values (1000-replicates UFBoot) are shown for all nodes. Eukaryotic sequence names are abbreviated with the four-letter code (see Table A in S1 Supporting information) and colored according to their major taxonomic group (see panel). All sequences starting with 'UP-' correspond to prokaryotic sequences. (PDF) [file pgen.1007986.s020.pdf]

Supplementary figure 17

NRT2 (euks, excluding Oomycota)

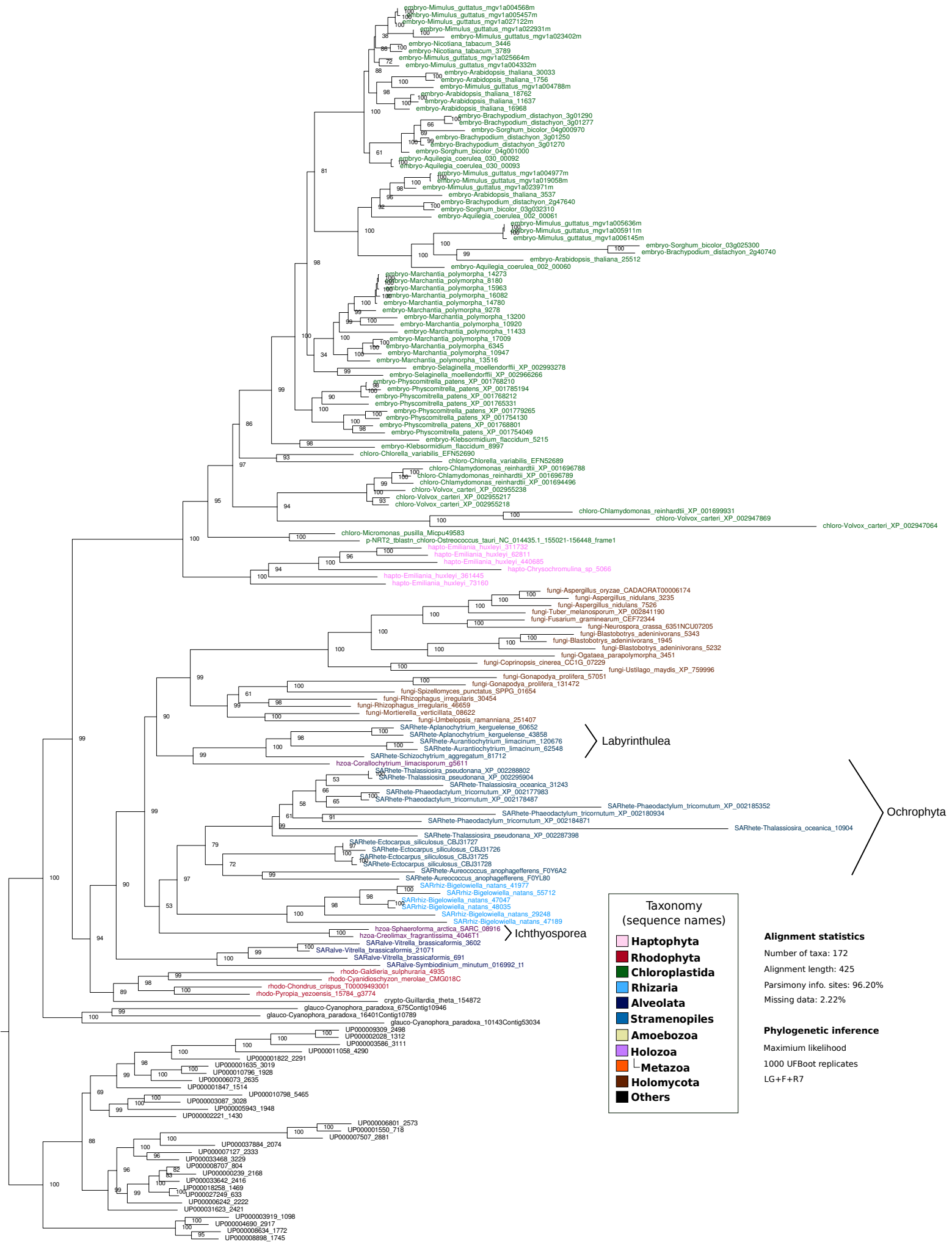

Supplement: S17 Fig — The tree was rooted at the branch that separates the eukaryotic clade from the bacterial sequences. Statistical support values (1000-replicates UFBoot) are shown in all nodes. Eukaryotic sequence names are abbreviated with the four-letter code (see Table A in S1 Supporting information) and colored according to their major taxonomic group (see panel). All sequences starting with 'UP-' correspond to prokaryotic sequences. (PDF) [file pgen.1007986.s021.pdf]

Supplementary figure 18

EUKNR (euks)

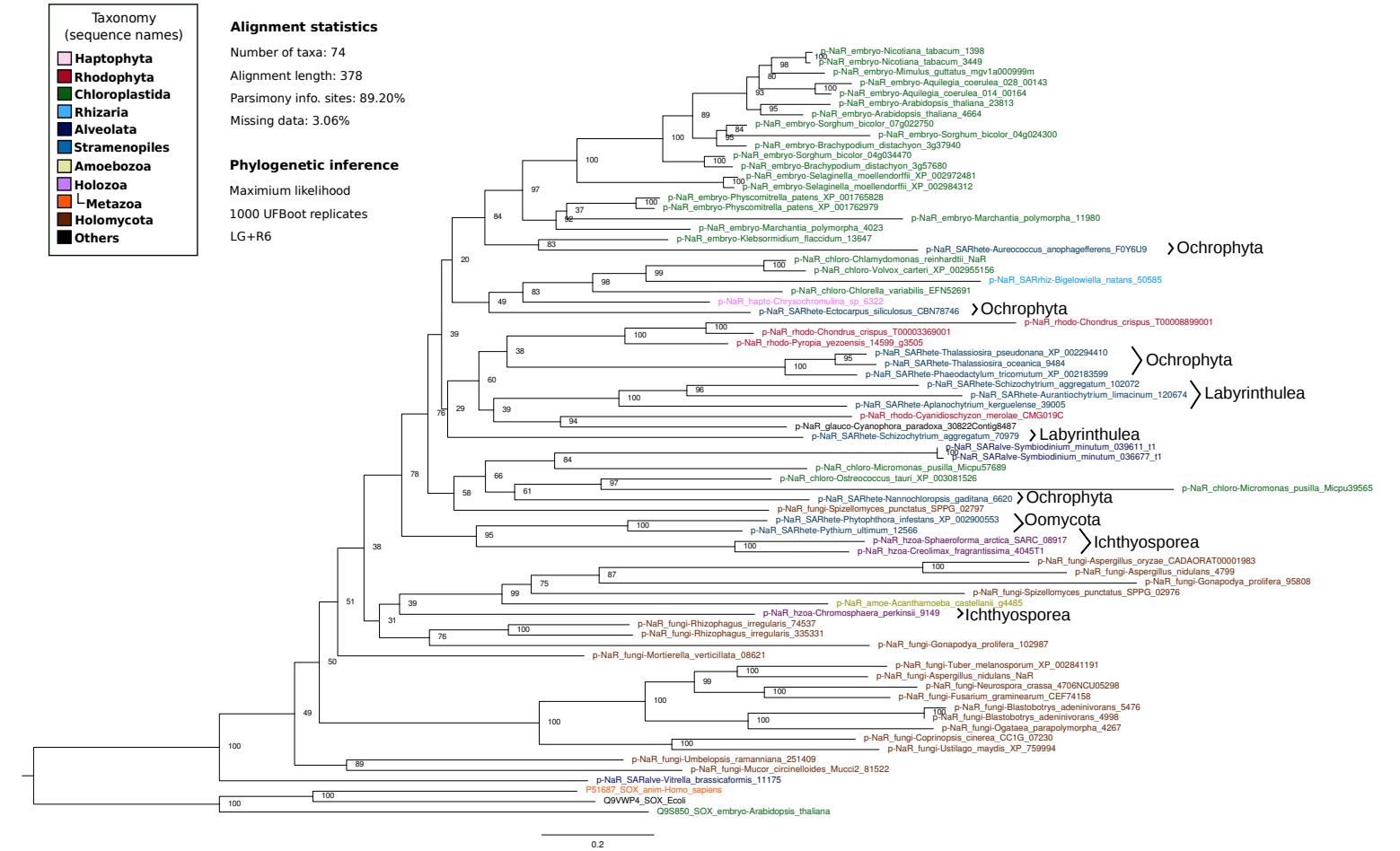

Supplement: S18 Fig — The tree was rooted in the branch that separates the EUKNR clade from the three sulfite oxidase sequences. Statistical support values (1000-replicates UFBoot) are shown in all nodes. Eukaryotic sequence names are abbreviated with the four-letter code (see Table A in S1 Supporting information) and colored according to their major taxonomic group (see panel). All sequences starting with 'UP-' correspond to prokaryotic sequences. (PDF) [file pgen.1007986.s022.pdf]

## TPmet tree (euks)

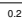

Supplement: S20 Fig — All the eukaryotic sequences of the tree are considered to belong to a subset of tetrapyrrole methylase proteins named TPmet family. Eukaryotic sequence names are abbreviated with the four-letter code (see Table A in S1 Supporting information) and colored according to their major taxonomic group (see panel). All sequences starting with 'UP-' correspond to prokaryotic sequences. The three sequences found in cluster with NAP genes are indicated with arrows. (PDF) [file pgen.1007986.s024.pdf]

**Supplementary figure 21**      TPmet distribution in relation to NAD(P)H-NIR

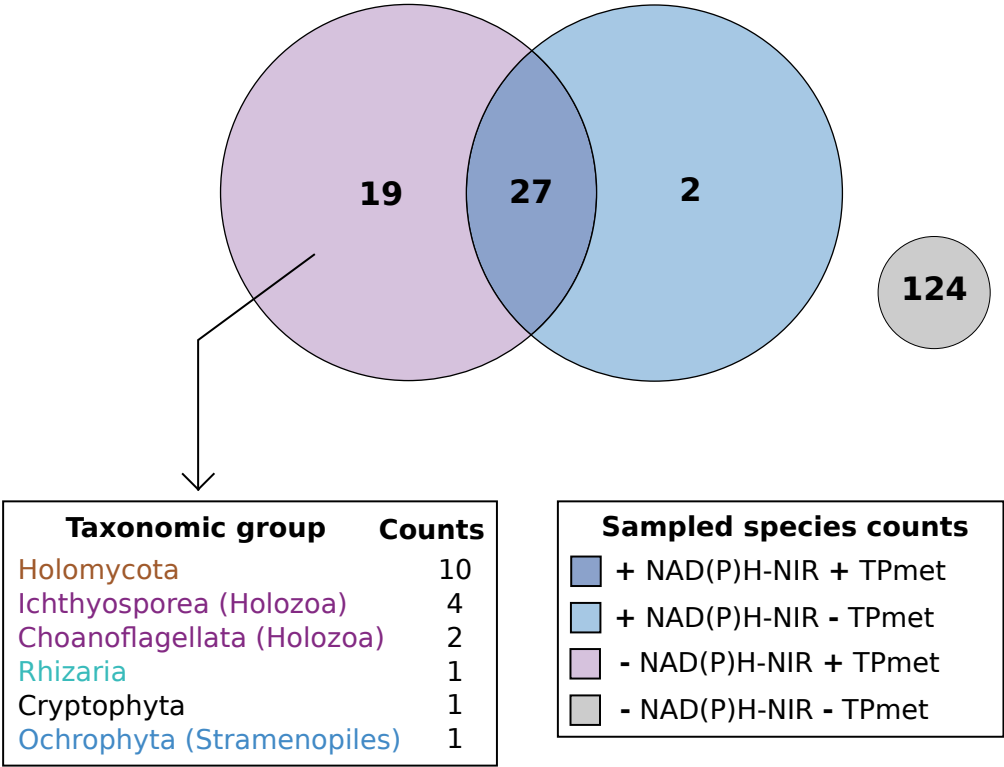

Supplement: S21 Fig — Venn diagram representing the quantitative distribution of the sampled eukaryotes (euk_db) recording the presence/absence of the NAD(P)H-nir and the TPmet genes. A ranking of the taxonomic groups that have at least one representative species with the TPmet but without the NAD(P)H-nir is also represented. (PDF) [file pgen.1007986.s025.pdf]

Supplementary figure S22

*Pyr\_redox\_2* and *Fer2\_BFD* Pfam domains tree  
(euks + proks + MMETSP + MDM\_db)

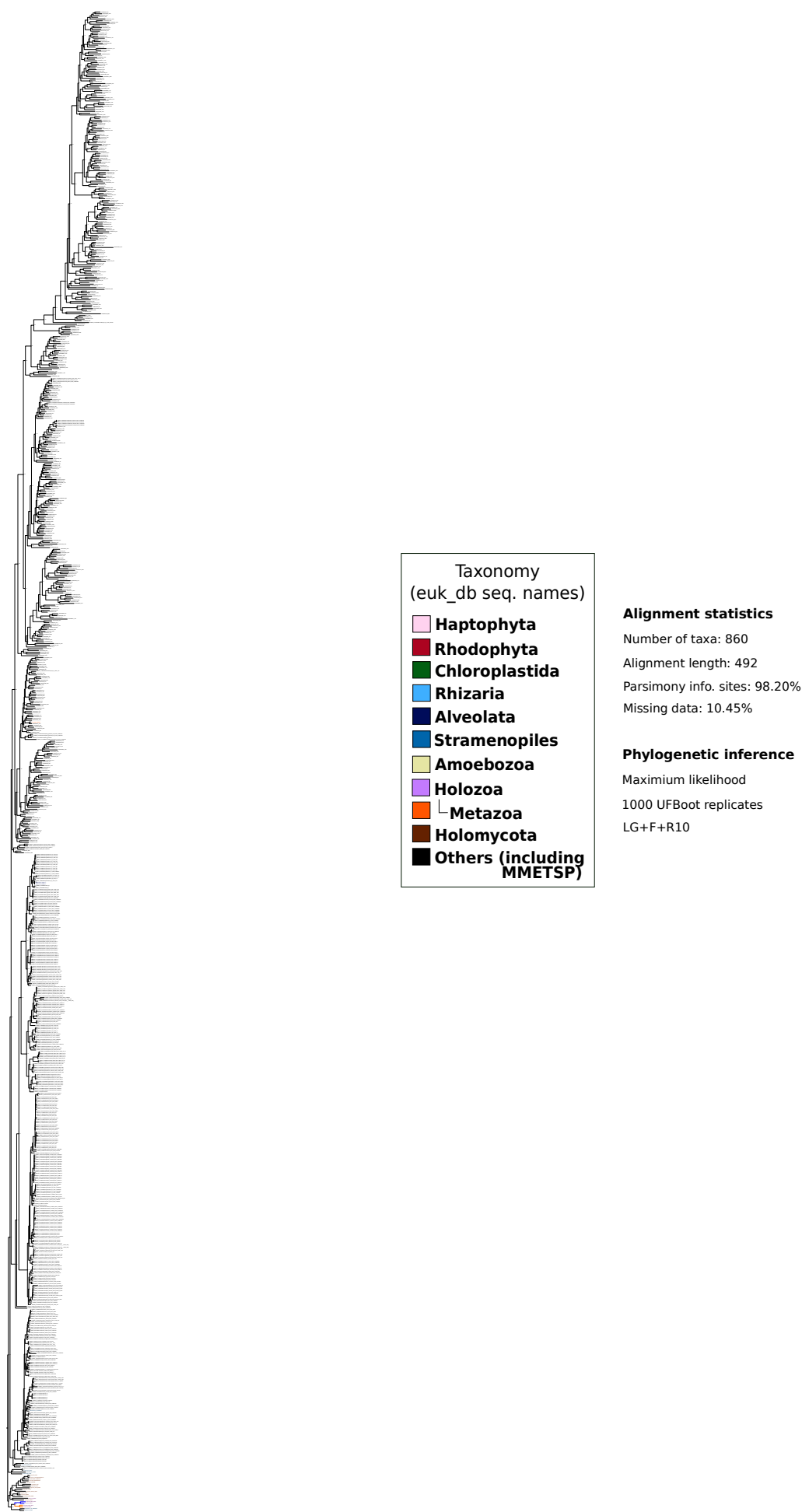

Supplement: S22 Fig — Eukaryotic sequence names are abbreviated with the four-letter code (see Table A in S1 Supporting information) and colored according to their major taxonomic group (see panel). All sequences starting with 'UP-' correspond to prokaryotic sequences. Sequences from MMETSP are colored in black. Blue and orange clades represent the sequences corresponding to the Creolimax fragrantissima and Sphaeroforma arctica EUKNR and NAD(P)H-NIR, respectively. (PDF) [file pgen.1007986.s026.pdf]

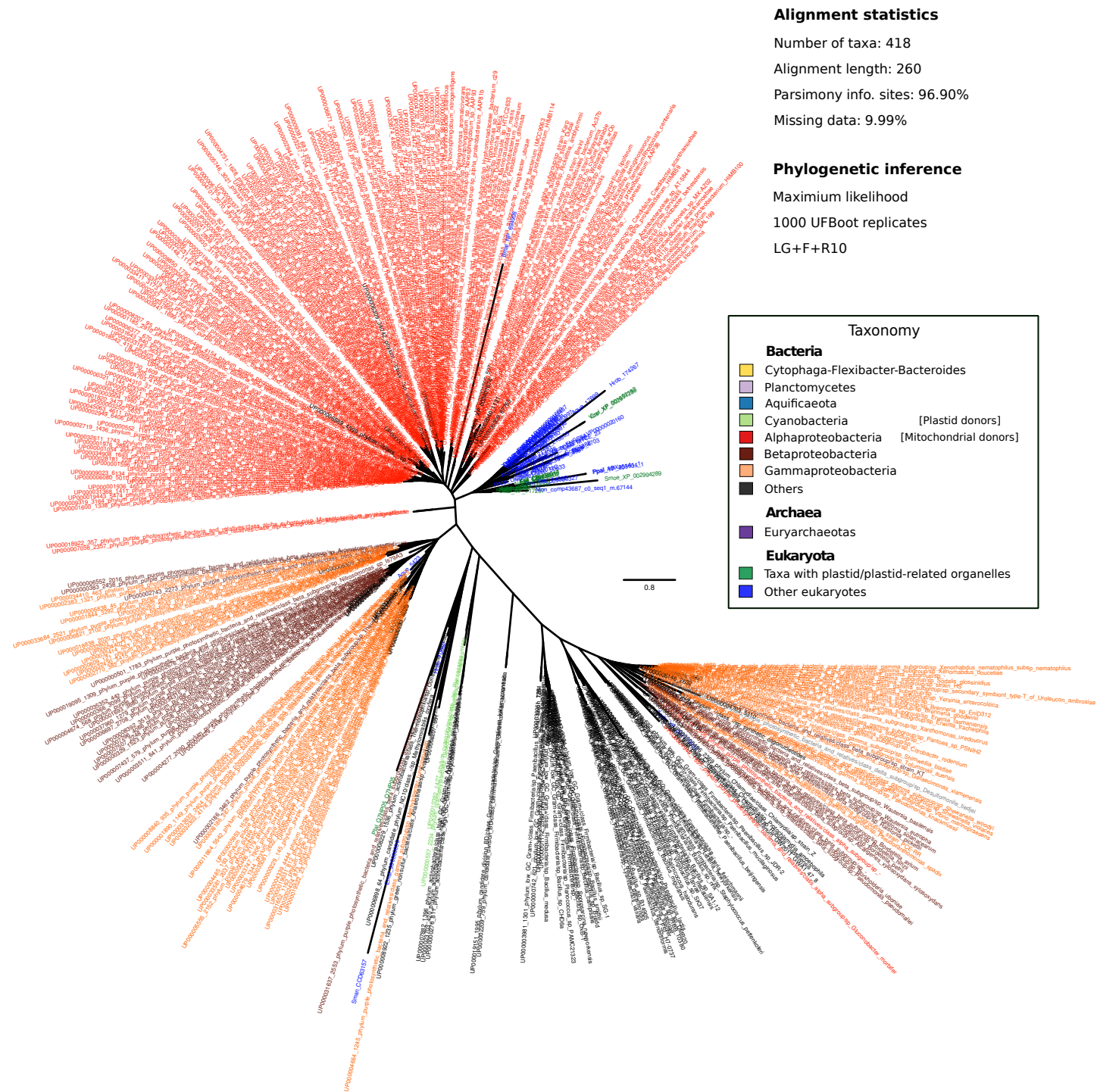

Supplement: S25 Fig — Prokaryotic sequences are colored according to the corresponding phylum or class, while eukaryotes are colored according to whether they contain or not a plastid/plastid-related organelle (see panel). As expected, Alphaproteobacteria is the sister group to eukaryotes, suggesting that the taxonomic representation of prok_db allow to detect proteins with signatures of Alphaproteobacteria, and hence of putative mitochondrial origin. The process of phylogenetic inference and taxonomic assignation is explained in Materials and methods section. (PDF) [file pgen.1007986.s029.pdf]

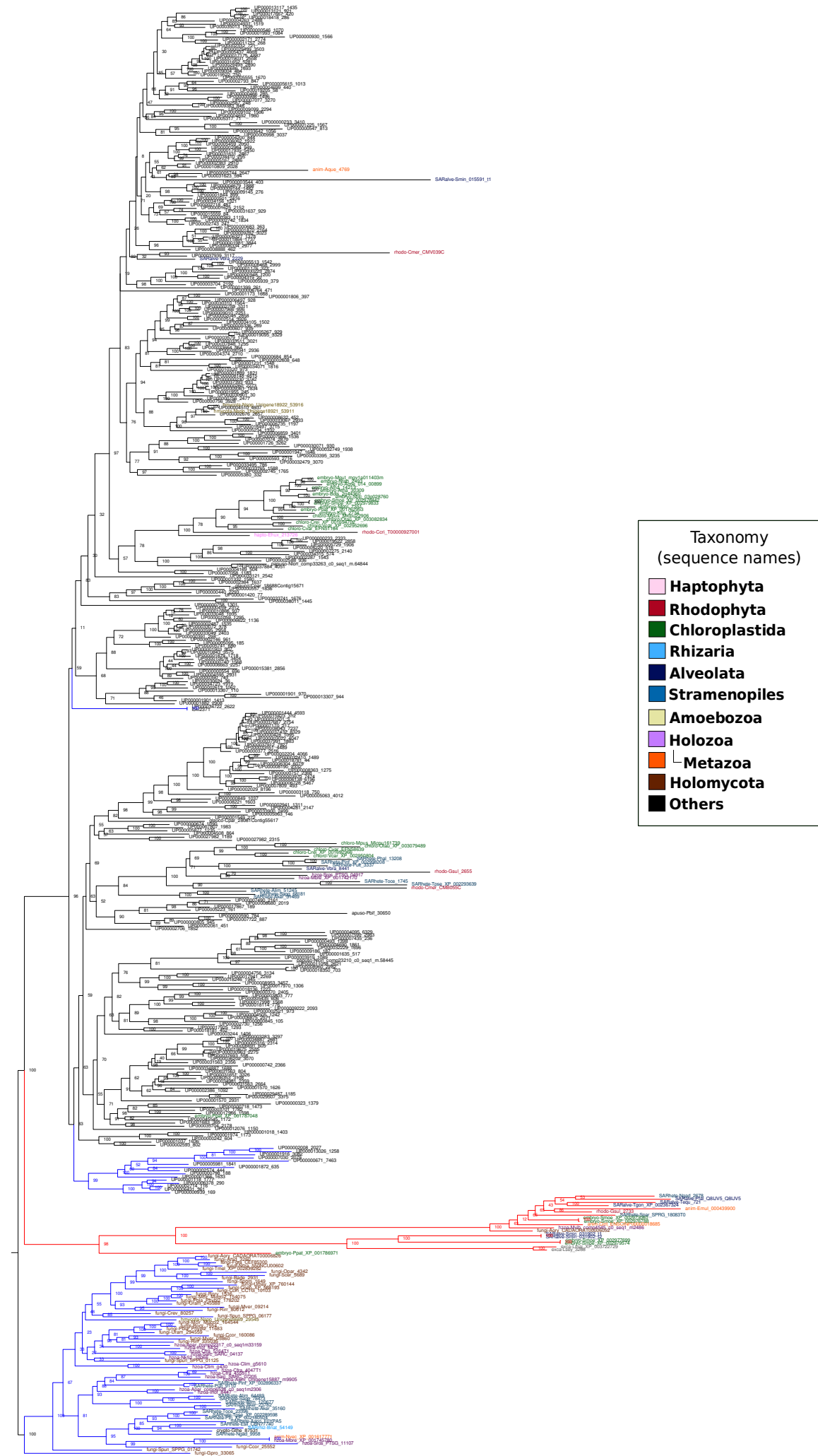

Supplement: S27 Fig — Eukaryotic sequence names are abbreviated with the four-letter code (see Table A in S1 Supporting information) and colored according to their major taxonomic group (see panel). All sequences starting with 'UP-' correspond to prokaryotic sequences. A third and last phylogenetic tree was constructed using sequences from the blue clades (see S20 Fig). (PDF) [file pgen.1007986.s031.pdf]
