# Supplementary material for: Reticulate evolution in eukaryotes: Origin and evolution of the nitrate assimilation pathway
Source: PLoS Genet. 2019 Feb 21;15(2):e1007986. doi: 10.1371/journal.pgen.1007986 (PMC6400420; doi:10.1371/journal.pgen.1007986)
Supplement: S2 Fig — (A) Correlation (from 0 to 1) between the distributions of the four NAPs in the entire eukaryotic dataset and (B) in eukaryotes from which at least one NAP was identified. (C) Correlation between the presence of NAPs with the nutrient acquisition strategies within the entire eukaryotic dataset (from 0 to 1) (see Materials and methods section). (PDF) [file pgen.1007986.s006.pdf]

Supplementary figure 2

Correlation between NAPs distribution and the different nutrient acquisition strategies

A) Correlation between NAPs distribution in the whole dataset

	■ *nrt2*	● *nr*	★ *nir-fd*	► *nir-NAD(P)H*
■ *nrt2*	1.00	0.94	0.82	0.84
● *nr*	0.94	1.00	0.82	0.85
★ *nir-fd*	0.82	0.82	1.00	0.73
► *nir-NAD(P)H*	0.84	0.85	0.73	1.00

B) Correlation between NAPs distribution, considering only taxa with at least 1 NAP

	■ *nrt2*	● *euknr*	★ *Fd-nir*	► *NAD(P)H-nir*
■ *nrt2*	1.00	0.81	0.49	0.53
● *euknr*	0.81	1.00	0.47	0.58
★ *Fd-nir*	0.49	0.47	1.00	0.22
► *NAD(P)H-nir*	0.53	0.58	0.22	1.00

C) Correlation between NAPs distribution and the different nutrient acquisition strategies

			■ *nrt2*	● *euknr*	★ *Fd-nir*	► *NAD(P)H-nir*
Autotrophs (70)			0.97	0.90	0.87	0.23
Non-autotrophs	Phagotrophs (65)		0.00	0.02	0.00	0.00
Non-autotrophs	Fungi-like osmotrophs	Fungi (31)	0.47	0.50	0.00	0.47
Non-autotrophs	Fungi-like osmotrophs	Teretosporea (9)	0.33	0.33	0.00	0.33
Non-autotrophs	Fungi-like osmotrophs	Oomycota (7)	0.71	0.29	0.00	0.29
Non-autotrophs	Fungi-like osmotrophs	Labyrinthulea (3)	1.00	1.00	0.00	1.00
Non-autotrophs	Others (26)		0.00	0.00	0.00	0.00

100%

0%
