# Supplementary material for: Reticulate evolution in eukaryotes: Origin and evolution of the nitrate assimilation pathway
Source: PLoS Genet. 2019 Feb 21;15(2):e1007986. doi: 10.1371/journal.pgen.1007986 (PMC6400420; doi:10.1371/journal.pgen.1007986)
Supplement: S5 Fig — Statistical support values (1000-replicates UFBoot) are shown for all nodes. Non-informative clades were collapsed. Prokaryotic sequences were colored according to the corresponding phylum or class, while eukaryotes were colored according to whether they contain or not a plastid/plastid-related organelle (see panel). (PDF) [file pgen.1007986.s009.pdf]

# Supplementary figure 5

## Ribosomal protein L1 (euks + proks)

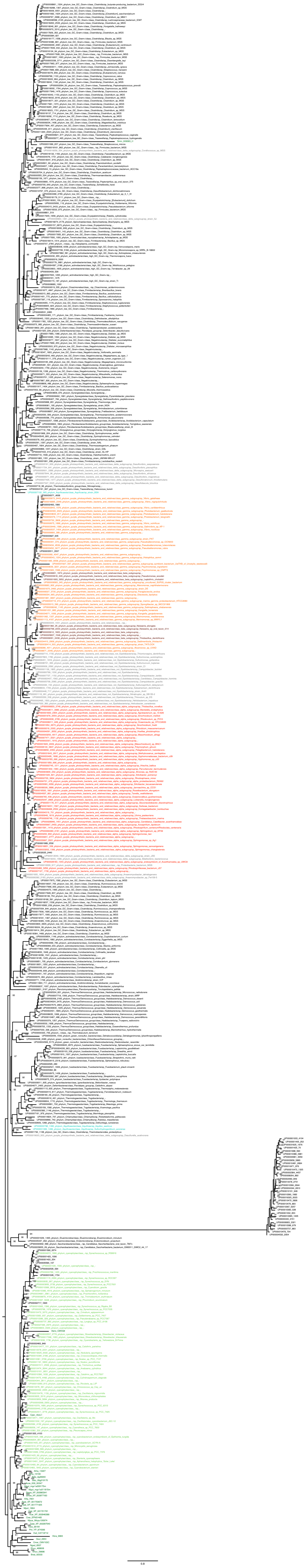

### Alignment statistics

Number of taxa: 500

Alignment length: 119

Parsimony info. sites: 96.60%

Missing data: 25%

### Phylogenetic inference

Maximium likelihood

1000 UFBoot replicates

LG+R8

### Taxonomy

**Bacteria**

- Cytophaga-Flexibacter-Bacteroides
- Planctomycetes
- Aquificaeota
- Cyanobacteria [Plastid donors]
- Alphaproteobacteria [Mitochondrial donors]
- Betaproteobacteria
- Gammaproteobacteria
- Others

**Archaea**

- Euryarchaeotas

**Eukaryota**

- Taxa with plastid/plastid-related organelles
- Other eukaryotes
