# Supplementary material for: Reticulate evolution in eukaryotes: Origin and evolution of the nitrate assimilation pathway
Source: PLoS Genet. 2019 Feb 21;15(2):e1007986. doi: 10.1371/journal.pgen.1007986 (PMC6400420; doi:10.1371/journal.pgen.1007986)
Supplement: S7 Fig — The tree was rooted in the branch that separates the eukaryotic clade from the rest of the tree. Statistical support values (1000-replicates UFBoot) are shown for all nodes. Prokaryotic sequences were colored according to the corresponding phylum or class, while eukaryotes were colored according to whether they contain or not a plastid/plastid-related organelle (see panel). (PDF) [file pgen.1007986.s011.pdf]

Supplementary figure 7

NRT2 (euks + proks)

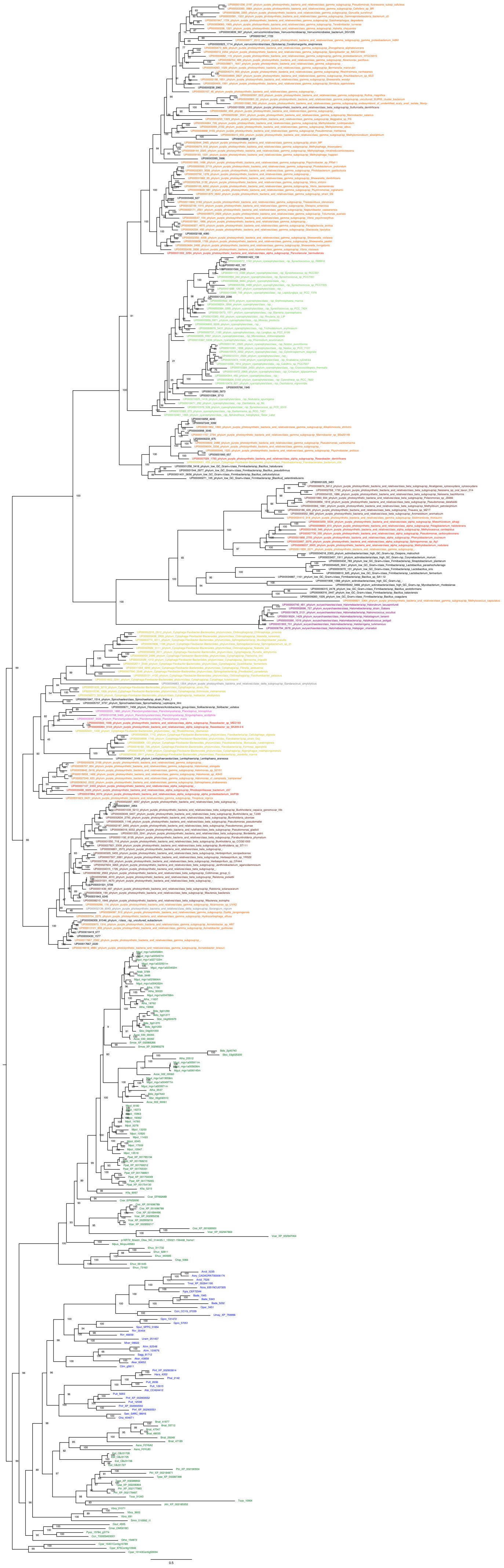

Alignment statistics

Number of taxa: 393

Alignment length: 441

Parsimony info. sites: 97.50%

Missing data: 6.09%

Phylogenetic inference

Maximium likelihood

1000 UFBoot replicates

LG+F+R10

Taxonomy

Bacteria

- Cytophaga-Flexibacter-Bacteroides (Phylum)
- Planctomycetes (Phylum)
- Aquificaeota (Phylum)
- Cyanophytes (Phylum) [Plastid donors]
- Purple photosynthetic bacteria and relatives (Phylum)
  - Alpha subgroup [Mitochondrial donors]
  - Beta subgroup
  - Gamma subgroup
  - Delta subgroup
- Others

Archaea

- Euryarchaeotas (Phylum)

Eukaryota

- Taxa with plastid/plastid-related organelles
- Other eukaroytes
