# Supplementary material for: Reticulate evolution in eukaryotes: Origin and evolution of the nitrate assimilation pathway
Source: PLoS Genet. 2019 Feb 21;15(2):e1007986. doi: 10.1371/journal.pgen.1007986 (PMC6400420; doi:10.1371/journal.pgen.1007986)
Supplement: S11 Fig — The tree was rooted in the branch that separates the eukaryotic clade from the bacterial sequences, with nodes. Statistical support values (1000-replicates UFBoot) are shown for all nodes. Eukaryotic sequence names are abbreviated with the four-letter code (see Table A in S1 Supporting information) and colored according to their major taxonomic group (see panel). All sequences starting with 'UP-' correspond to prokaryotic sequences. (PDF) [file pgen.1007986.s015.pdf]

Supplementary figure 11 NAD(P)H-NIR (euks, excluding Ichthyosporea)

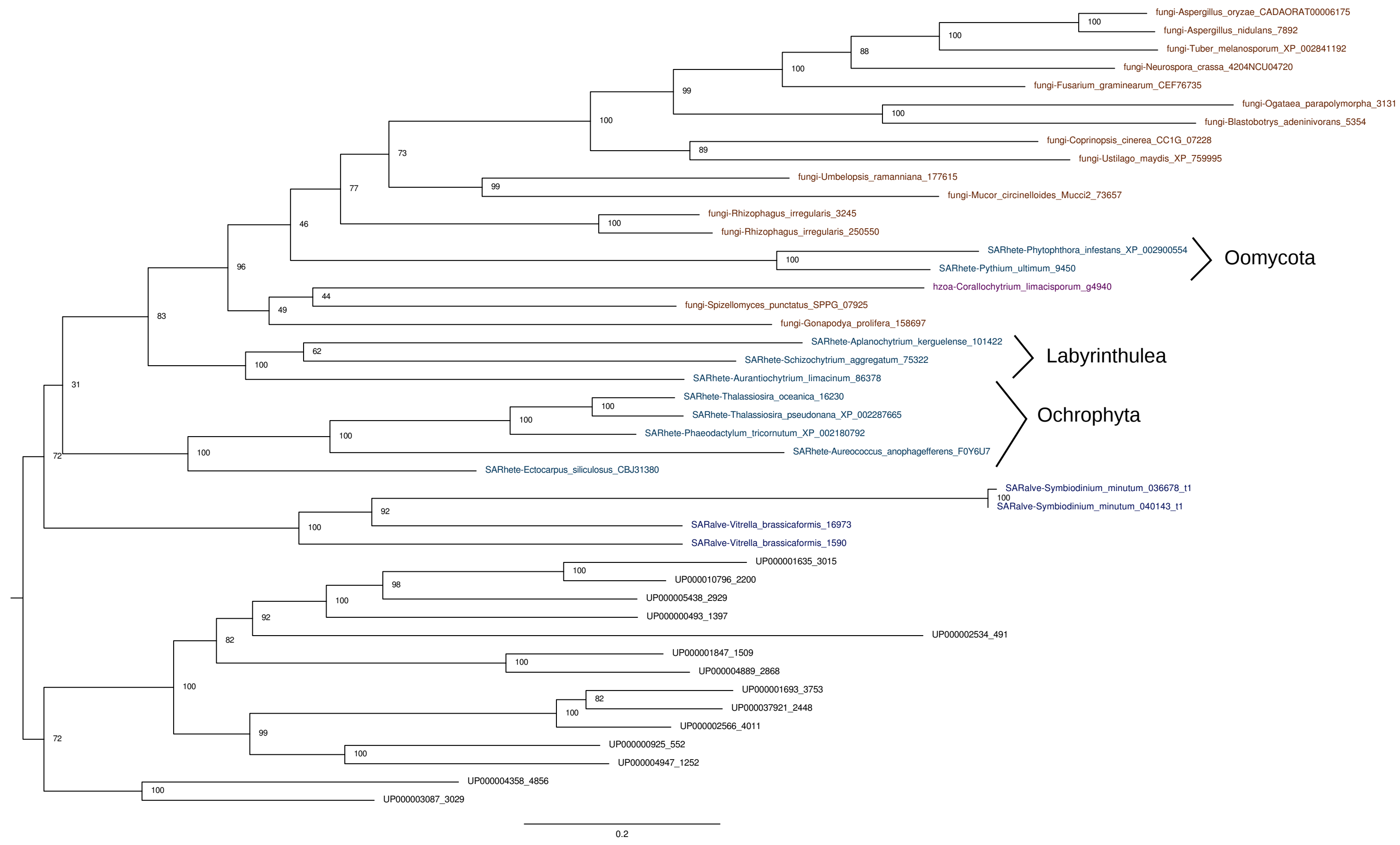

Alignment statistics

Number of taxa: 44

Alignment length: 805

Parsimony info. sites: 79.6%

Missing data: 1.30%

Phylogenetic inference

Maximium likelihood

1000 UFBoot replicates

LG+R5

Taxonomy
(sequence names)

- **Haptophyta**
- **Rhodophyta**
- **Chloroplastida**
- **Rhizaria**
- **Alveolata**
- **Stramenopiles**
- **Amoebozoa**
- **Holozoa**
  - **Metazoa**
- **Holomycota**
- **Others**
