# Supplementary material for: Reticulate evolution in eukaryotes: Origin and evolution of the nitrate assimilation pathway
Source: PLoS Genet. 2019 Feb 21;15(2):e1007986. doi: 10.1371/journal.pgen.1007986 (PMC6400420; doi:10.1371/journal.pgen.1007986)
Supplement: S19 Fig — Eukaryotic sequence names are abbreviated with the four-letter code (see Table A in S1 Supporting information) and colored according to their major taxonomic group (see panel). All sequences starting with 'UP-' correspond to prokaryotic sequences. A second phylogenetic tree (S27 Fig) was constructed using sequences from the blue clade (named as TPmet proteins, see Materials and methods section). The three sequences found in cluster with NAP genes are indicated with arrows. (PDF) [file pgen.1007986.s023.pdf]

Supplementary figure 19

TP_methylase draft tree (euks + proks)

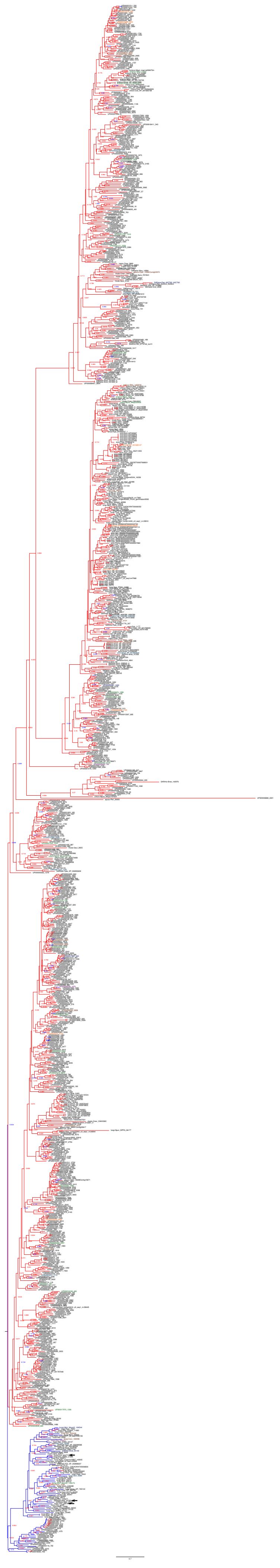

Taxonomy
(sequence names)

- **Haptophyta**
- **Rhodophyta**
- **Chloroplastida**
- **Rhizaria**
- **Alveolata**
- **Stramenopiles**
- **Amoebozoa**
- **Holozoa**
  - └ **Metazoa**
- **Holomycota**
- **Others**

Alignment statistics

Number of taxa: 1057

Alignment length: 147

Parsimony info. sites: 99.30%

Missing data: 1.8%

Phylogenetic inference

Maximium likelihood

FastTree default bootstrap

Jukes-Cantor+CAT
