# Supplementary material for: Reticulate evolution in eukaryotes: Origin and evolution of the nitrate assimilation pathway
Source: PLoS Genet. 2019 Feb 21;15(2):e1007986. doi: 10.1371/journal.pgen.1007986 (PMC6400420; doi:10.1371/journal.pgen.1007986)

Supplementary figure S23 NRT2 (euks, excluding Labyrinthulea and Ichthyosporea)

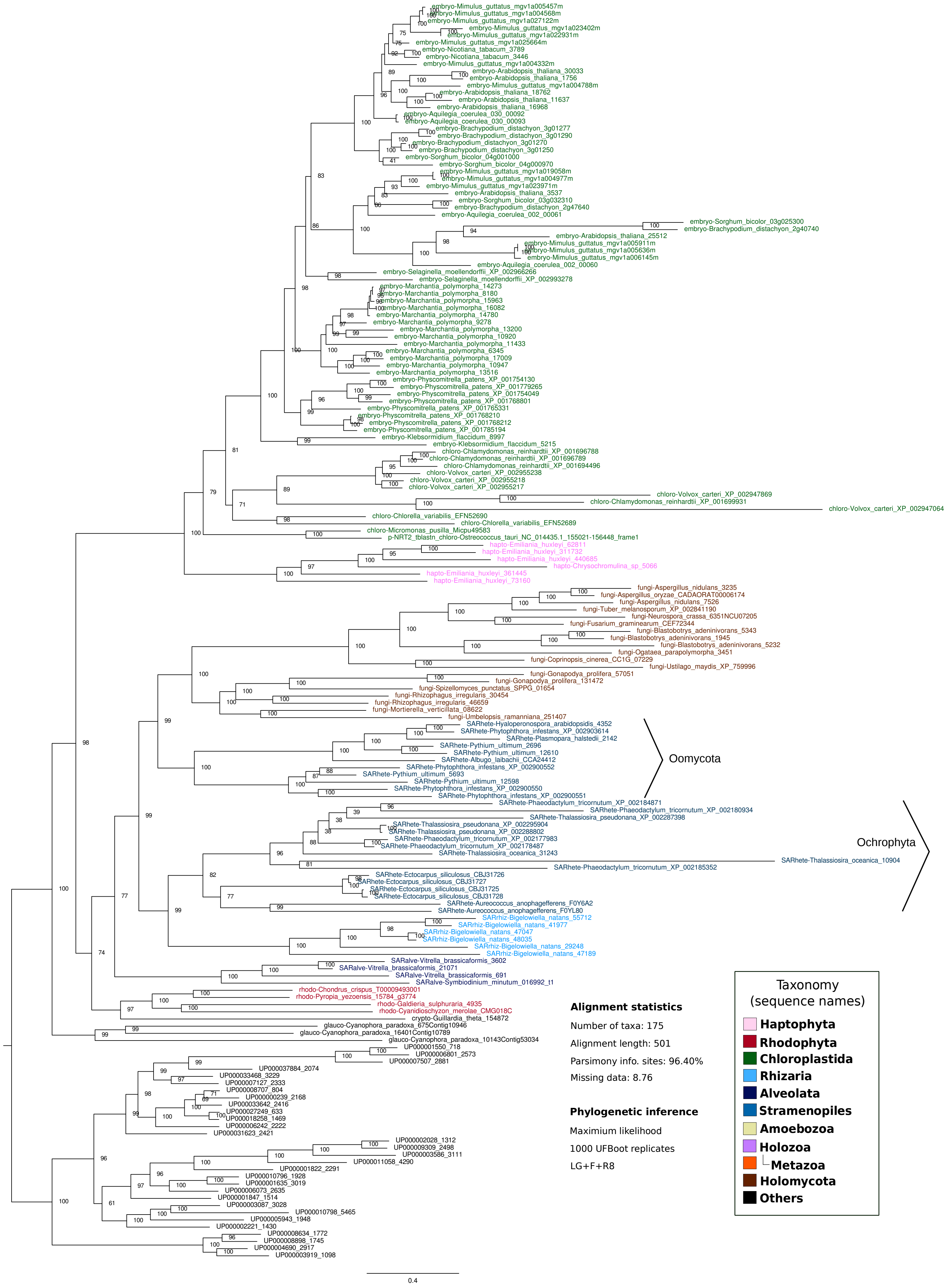

Supplement: S23 Fig — The tree was rooted at the branch that separates the eukaryotic clade from the bacterial sequences. Statistical support values (1000-replicates UFBoot) are shown in all nodes. Eukaryotic sequence names are abbreviated with the four-letter code (see Table A in S1 Supporting information) and colored according to their major taxonomic group (see panel). All sequences starting with 'UP-' correspond to prokaryotic sequences. (PDF) [file pgen.1007986.s027.pdf]
