# Supplementary material for: Reticulate evolution in eukaryotes: Origin and evolution of the nitrate assimilation pathway
Source: PLoS Genet. 2019 Feb 21;15(2):e1007986. doi: 10.1371/journal.pgen.1007986 (PMC6400420; doi:10.1371/journal.pgen.1007986)
Supplement: S24 Fig — The tree was rooted in the branch that separates the eukaryotic clade from the bacterial sequences, with nodes. Statistical support values (1000-replicates UFBoot) are shown for all nodes. Eukaryotic sequence names are abbreviated with the four-letter code (see Table A in S1 Supporting information) and colored according to their major taxonomic group (see panel). All sequences starting with 'UP-' correspond to prokaryotic sequences. (PDF) [file pgen.1007986.s028.pdf]

Supplementary figure S24

NAD(P)H-NIR (euks, excluding Labyrinthulea and Ichthyosporea)

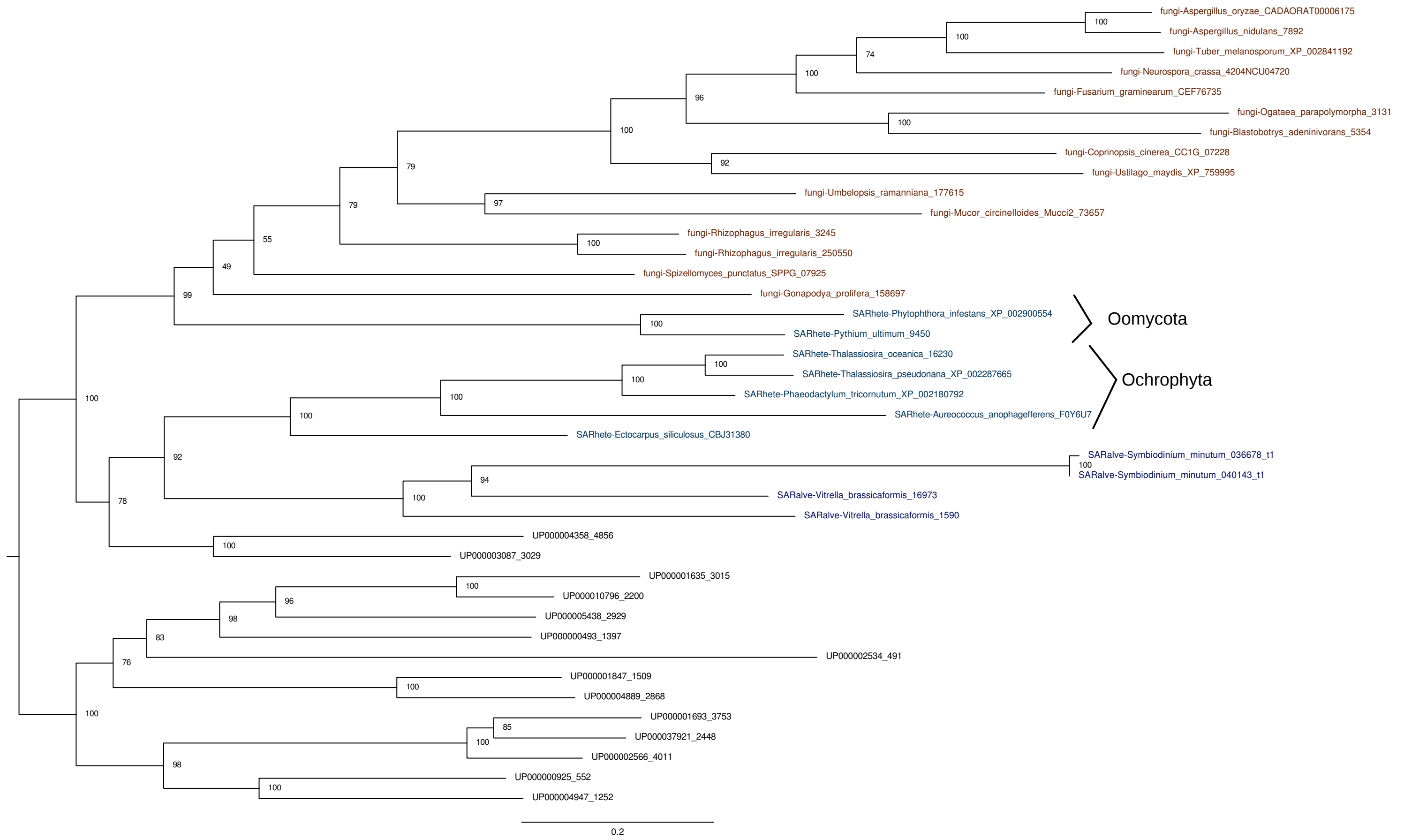

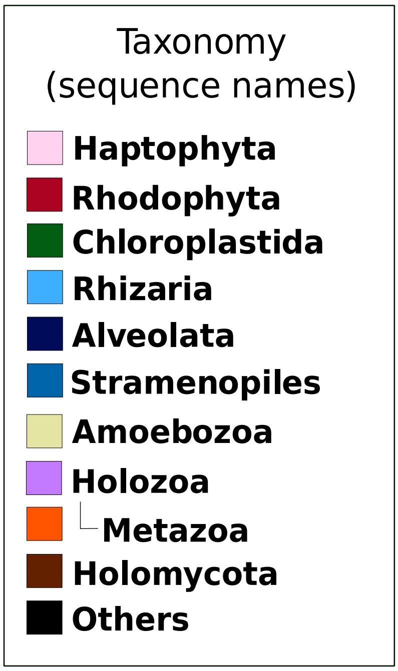

Alignment statistics

Number of taxa: 40

Alignment length: 807

Parsimony info. sites: 78.70%

Missing data: 1.43%

Phylogenetic inference

Maximium likelihood

1000 UFBoot replicates

LG+R5
