# Supplementary material for: Reticulate evolution in eukaryotes: Origin and evolution of the nitrate assimilation pathway
Source: PLoS Genet. 2019 Feb 21;15(2):e1007986. doi: 10.1371/journal.pgen.1007986 (PMC6400420; doi:10.1371/journal.pgen.1007986)
Supplement: S26 Fig — Prokaryotic sequences are colored according to the corresponding phylum or class, while eukaryotes are colored according to whether they contain or not a plastid/plastid-related organelle (see panel). As expected, Alphaproteobacteria is the sister group to eukaryotes, suggesting that the taxonomic representation of prok_db allow to detect proteins with signatures of Alphaproteobacteria, and hence of putative mitochondrial origin. The process of phylogenetic inference and taxonomic assignation is explained in Materials and methods section. (PDF) [file pgen.1007986.s030.pdf]

Supplementary figure S26

Cyt-B (euks & proks)

Alignment statistics

Number of taxa: 473

Alignment length: 754

Parsimony info. sites: 85.90%

Missing data: 50.68%

Phylogenetic inference

Maximium likelihood

1000 UFBoot replicates

LG+R10

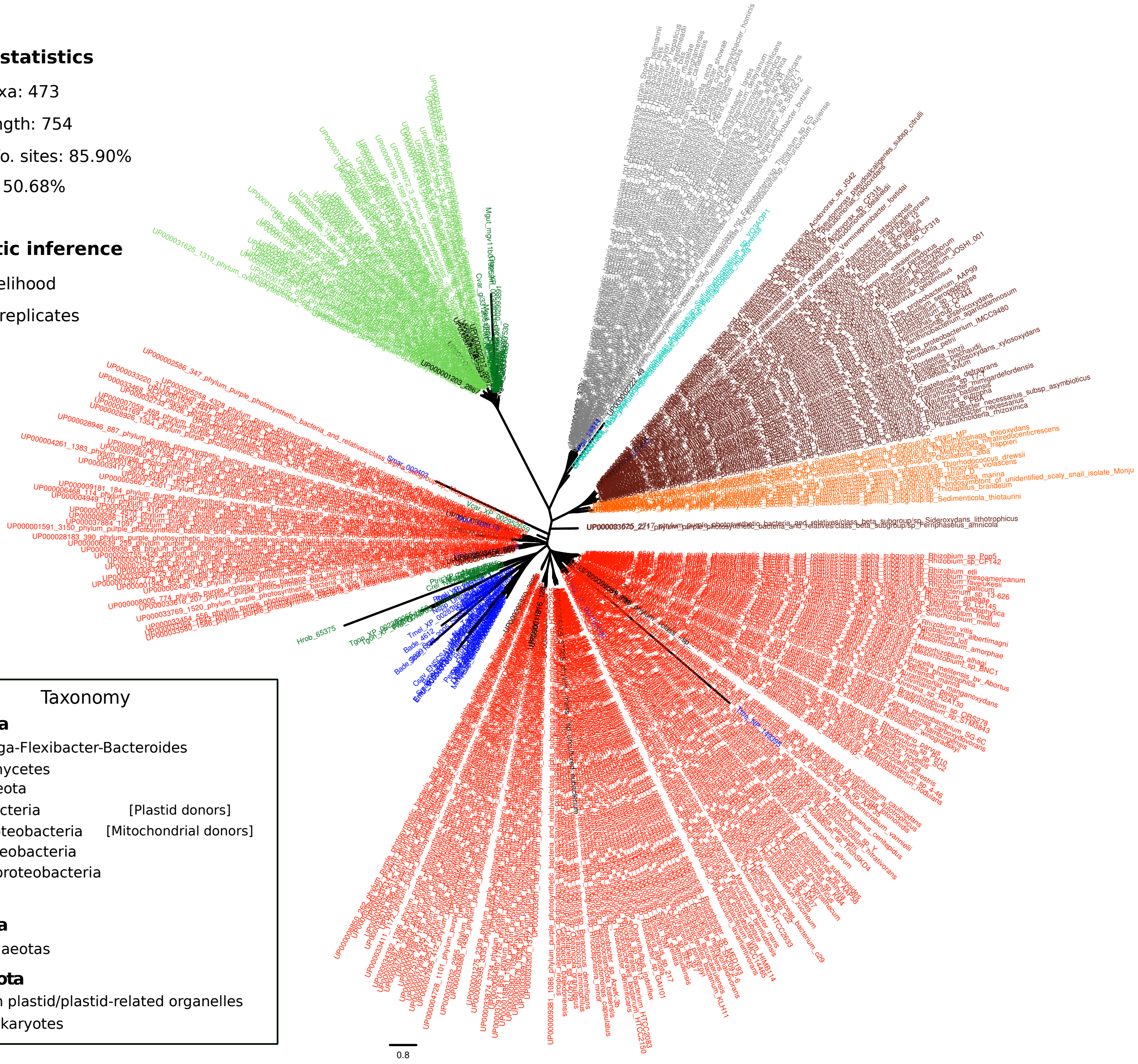

Taxonomy

Bacteria

- Cytophaga-Flexibacter-Bacteroides
- Planctomycetes
- Aquificaeota
- Cyanobacteria [Plastid donors]
- Alphaproteobacteria [Mitochondrial donors]
- Betaproteobacteria
- Gammaproteobacteria
- Others

Archaea

- Euryarchaeotas

Eukaryota

- Taxa with plastid/plastid-related organelles
- Other eukaryotes
